# Genetic and endosymbiotic diversity of Greek populations of *Philaenus spumarius*, *Philaenus signatus* and *Neophilaenus campestris*, vectors of *Xylella fastidiosa*

**DOI:** 10.1038/s41598-021-83109-z

**Published:** 2021-02-12

**Authors:** Despoina Ev. Kapantaidaki, Spyridon Antonatos, Vasiliki Evangelou, Dimitrios P. Papachristos, Panagiotis Milonas

**Affiliations:** Scientific Directorate of Entomology and Agricultural Zoology, Benaki Phytopathological Institute, 8 St. Delta str., Kifissia, Attica, Greece

**Keywords:** Symbiosis, Entomology, Population genetics, Haplotypes, Genetic variation, DNA sequencing, Symbiosis

## Abstract

The plant-pathogenic bacterium *Xylella fastidiosa* which causes significant diseases to various plant species worldwide, is exclusively transmitted by xylem sap-feeding insects. Given the fact that *X. fastidiosa* poses a serious potential threat for olive cultivation in Greece, the main aim of this study was to investigate the genetic variation of Greek populations of three spittlebug species (*Philaenus spumarius*, *P. signatus* and *Neophilaenus campestris*), by examining the molecular markers Cytochrome Oxidase I, cytochrome b and Internal Transcribed Spacer. Moreover, the infection status of the secondary endosymbionts *Wolbachia*, *Arsenophonus*, *Hamiltonella*, *Cardinium* and *Rickettsia*, among these populations, was determined. According to the results, the *ITS2* region was the less polymorphic, while the analyzed fragments of *COI* and *cytb* genes, displayed high genetic diversity. The phylogenetic analysis placed the Greek populations of *P. spumarius* into the previously obtained Southwest clade in Europe. The analysis of the bacterial diversity revealed a diverse infection status. *Rickettsia* was the most predominant endosymbiont while *Cardinium* was totally absent from all examined populations. *Philaenus spumarius* harbored *Rickettsia*, *Arsenophonus*, *Hamiltonella* and *Wolbachia*, *N. campestris* carried *Rickettsia*, *Hamiltonella* and *Wolbachia* while *P. signatus* was infected only by *Rickettsia*. The results of this study will provide an important knowledge resource for understanding the population dynamics of vectors of *X. fastidiosa* with a view to formulate effective management strategies towards the bacterium.

## Introduction

*Xylella fastidiosa* is a notorious xylem-inhabiting, vector-transmitted, Gram-negative bacterium^1^. It causes economically important diseases to crop, forest and landscape plants, such as Pierce’s disease of grapevine, leaf scorch of almond, oleander and coffee, citrus variegated chlorosis and phony peach disease^2^. It is exclusively transmitted by xylem sap-feeding insects belonging to the order Hemiptera, sub-order Auchenorrhyncha^3^. In Europe, it has been described for the first time in south Italy causing the olive quick decline syndrome^4^. The main vector in Europe is considered the spittlebug *Philaenus spumarius* (Hemiptera: Aphrophoridae), although other species have been proven to be able to transmit the bacterium^5^. In olive orchards the most abundant species are *P. spumarius* and *Neophilaenus campestris*^6–8^. *Philaenus signatus* (Hemiptera: Aphrophoridae) has also been found in Greek olive orchards, in lower densities though^9^.

The recent phylogeographic and phylogenetic studies on the population genetics of the genus *Philaenus* were focused on the widely distributed species *P. spumarius* which is one of the most well studied Auchenorrhyncha^10–15^. The biogeographical pattern of the species based on the combination of three mitochondrial genes (*COI*, *COII*, *cytb*) presents the existence of two main mtDNA lineages; a. the northeastern (NE) or eastern centered in Anatolia/Caucasus, from where the species expanded to north and central Europe and b. the southwestern (SW) or western centered in the Mediterranean region^11,12,14^. The existence of two clades was attributed to the bacterium *Wolbachia* that could possibly provoke speciation since individuals harboring it, belonged mainly to the NE clade^15^, and only single infections were presented in individuals from the SW clade.

Symbiosis between insects and bacteria is a common long-term phenomenon^16,17^. The obligate (primary) endosymbionts which exhibit 100% infection frequencies in hosts populations usually supplement the hosts’ imbalanced diets through the provision of essential nutrients such as amino acids^18^. The facultative (secondary) endosymbionts, which are not essential for their hosts, exhibit multiple infections from one or more endosymbionts of the same or different supergroups and strains and diverse infection frequencies, depending on the endosymbiont and insect host species, the environmental conditions, the host plant etc.^17,19^. To ensure their maintenance in host populations, they have gained the ability to cause important effects on their insect hosts on various aspects of their biology and evolutionary processes^17,20^.

The hemipteran sap-feeding insects of the suborder Auchenorrhyncha (e.g. leafhoppers, spittlebugs, cicadas) bring along more than one bacterial endosymbiont^16,21,22^. Generally, although studies elucidating different aspects on the obligatory endosymbionts^23^ exist, the facultative bacterial species of spittlebugs are less known, regardless their importance and impacts on hosts ‘biology, ecology and evolution’.

To shed light on the evolutionary processes of insect pests and to develop effective and successful management programs, it is critical to identify and understand their genetic variation and phenotypic variability (host spectrum and nutritional ecology, fitness, adaptation to different environments and invasiveness, heat tolerance, pathogen and parasite resistance) which are strongly influenced by the symbiotic communities they carry^17,18^.

In this framework, the main objective of this study was to assess the molecular genetic variation among and within three spittlebug species (*P. spumarius*, *P. signatus* and *N. campestris*), vectors of *X. fastidiosa*, by using mitochondrial and nuclear markers, in populations collected from different olive growing areas of Greece. Moreover, we investigated the distribution and infection status from five secondary endosymbionts (*Wolbachia*, *Arsenophonus*, *Hamiltonella*, *Cardinium*, *Rickettsia*), among these populations. From the data obtained, the correlation between the identified patterns of bacterial infection and of genetic polymorphism as well as the possible role of endosymbionts in the three species, is discussed.

## Results

The molecular analysis for the detection of *X. fastidiosa*, revealed the absence of the bacterium in all 279 individuals used in this study, while positive control templates produced fragments of the expected size. Negative control templates gave no amplification products.

### Diversity and genetic analysis

In the 87, 86 and 87 individuals of *P. spumarius* analyzed, there were a total of 20, 41 and 4 different haplotypes for COI, cytb, and ITS2 molecular markers, respectively. The total number of different haplotypes identified for *COI*, *cytb* and *ITS2*, were 3, 6 and 1, among 23, 18 and 24 individuals of *P. signatus*. The 47, 59 and 62 individuals of *N. campestris* analyzed revealed the existence of 13, 23 and 4 different haplotypes for *COI*, *cytb* and *ITS2*, respectively (Table 1 and Supplementary Table S1). Most populations were polymorphic, carrying more than one haplotype, with most of them being presented in one or a few individuals from the same population.

**Table 1:** Total number of individuals within each population of the three insect-species examined for genetic analysis and total number of haplotypes detected within each population, for each molecular marker analyzed.

Insect species	Population code	*COI*		*cytb*		*ITS2*	
		Number of individuals analyzed	No of different haplotypes detected	Number of individuals analyzed	No of different haplotypes detected	Number of individuals analyzed	No of different haplotypes detected
*P. spumarius*	570	12	8	10	5	12	1
*P. spumarius*	545	10	2	10	10	9	1
*P. spumarius*	540	10	2	10	6	8	1
*P. spumarius*	539	4	2	5	4	5	1
*P. spumarius*	546	8	3	10	7	9	1
*P. spumarius*	548	10	4	10	5	10	2
*P. spumarius*	547	10	6	10	10	9	1
*P. spumarius*	642	12	4	9	1	12	3
*P. spumarius*	643	11	3	12	6	13	2
*P. signatus*	543	7	3	6	3	7	1
*P. signatus*	580	6	2	4	2	7	1
*P. signatus*	645	10	2	8	5	10	1
*N. campestris*	542	15	3	16	11	17	3
*N. campestris*	541	8	5	10	7	11	2
*N. campestris*	556	14	8	20	9	20	3
*N. campestris*	644	10	4	13	6	14	2

The new sequences obtained for the three molecular markers in the three insect species, were deposited in GenBank database (http://www.ncbi.nlm.nih.gov/genbank/), under the following Accession Numbers: *P. spumarius* (*COI*: MT434012-MT434031, *cytb*: MT433054-MT433092, *ITS2*: MT434752-MT434755), *P. signatus* (*COI*: MT434032-MT434034, *cytb*: MT433025-MT433030, *ITS2*: MT434756) and *N. campestris* (*COI*: MT434035-MT434047, *cytb*: MT433031-MT433053, *ITS2*: MT434698-MT434701).

In Supplementary Table S2, genetic indices for *COI*, *cytb* and *ITS2* sequences derived from *P. spumarius, P. signatus and N. campestris* populations, collected from the eight Regional Units, are shown. The *ITS2* region was the less polymorphic in all three insect species, while the analyzed fragments of *COI* and *cytb* mitochondrial genes, displayed higher values of genetic diversity, mostly due to point mutations (Supplementary Tables S1 and S2).

A total of 42 and 21 polymorphic sites and 11 and 8 parsimony informative sites were found in *P. spumarius* populations for *cytb* and *COI* respectively. The separation of populations according to Regional Units revealed similar levels of genetic (haplotype and nucleotide) diversities, except for the population from Chania (South Greece) for the *COI* gene, which was less variable (0.200 ± 0.154 and 0.00087 ± 0.00067, haplotype and nucleotide diversity, respectively) (Supplementary Table S2). Haplotype diversity for cytb was very high (Hd: 1 ± 0.045) in the populations collected from North Athens and Kavala (North Greece), as all individuals of each of the two populations, had unique haplotypes (Supplementary Table S2). When the populations of *P. spumarius* were clustered into three groups according to their collected region (North, Central and South Greece), significant differences were not observed (Supplementary Table S2). North Greece which contained the population from Kavala (Table 2) displayed slightly higher genetic values for both *COI* and *cytb* genes than the other two groups (Central and South Greece) (Supplementary Table S2).

**Table 2:** Percentages of specimens within each population for the three insect species infected with secondary endosymbionts (*Arsenophonus*, *Halmitonella*, *Rickettsia*, *Cardinium* and *Wolbachia*).

Insect species	Population code	Regional Unit	Sampling site	Total number of individuals	% infection—*Arsenophonus*	% infection—*Hamiltonella*	% infection—*Rickettsia*	% infection – *Cardinium*	% infection—*Wolbachia*
*P. spumarius*	570	East Attica	Spata	23	0	0	78.26 (18)	0	4.35 (1)
*P. spumarius*	545	North Athens	Kifissia	21	0	4.76 (1)	0	0	4.76 (1)
*P. spumarius*	540	Chania	Vatolakos	11	0	36.36 (4)	45.45 (5)	0	0
*P. spumarius*	539	Cephalonia	Damoulianata	5	0	0	100 (5)	0	0
*P. spumarius*	546	Aetolia-Acarnania	Kandila	20	0	10 (2)	0	0	10 (2)
*P. spumarius*	548	Aetolia-Acarnania	Floriada	20	20 (4)	15 (3)	0	0	15 (3)
*P. spumarius*	547	Kavala	Mirtofito	21	14.28 (3)	0	4.76 (1)	0	0
*P. spumarius*	642	Achaea	Lakopetra	20	0	10 (2)	45 (9)	0	0
*P. spumarius*	643	Corinthia	Ancient Korinthos	20	0	0	5	0	0
*P. signatus*	543	Cephalonia	Livadi	10	0	0	70 (7)	0	0
*P. signatus*	580	Cephalonia	Damoulianata	16	0	0	93.75 (15)	0	0
*P. signatus*	645	Corinthia	Ancient Korinthos	10	0	0	100 (10)	0	0
*N. campestris*	542	East Attica	Vravrona	20	0	10 (2)	95 (19)	0	85 (17)
*N. campestris*	541	East Attica	Spata	20	0	25 (5)	50 (10)	0	60 (12)
*N. campestris*	556	Corinthia	Ancient Korinthos	21	0	4.76 (1)	85.71 (18)	0	19.04 (4)
*N. campestris*	644	Achaea	Lakopetra	20	0	5 (1)	5 (1)	0	0

The number in the parentheses indicates the number of individuals found harboring the corresponding endosymbiont.

Populations of *N. campestris,* displayed 22 and 10 polymorphic sites and 10 and 7 parsimony informative sites for *cytb* and *COI*, respectively (Supplementary Table S2). Populations from all Regional Units were found to be polymorphic and exhibited similar levels of genetic diversity for *COI* and *cytb* genes, but with an exception. Population from East Attica_Vravrona was shown to be genetically more diverse in the *cytb* gene (Hd: 0.933 ± 0.048), comparing to the *COI* gene for which the variance of haplotype diversity was the lowest (Hd: 0.257 ± 0.142) (Supplementary Table S2).

*Philaenus signatus* populations displayed lower genetic diversity, than that of the other two insect species (6 and 3, and 2 and 3, polymorphic and parsimony informative sites, for *cytb* and *COI*, respectively) (Supplementary Table S2). The pattern of variation of nucleotide diversity was congruent with the pattern of haplotype diversity in *COI* gene. On the other hand, the higher point estimates of haplotype diversity and the low nucleotide diversity values found in *cytb* gene, indicated that these populations might have undergone a recent population expansion after a period of low effective population size.

### Genetic structure of Greek populations of *P. spumarius*

**Figure 1:**
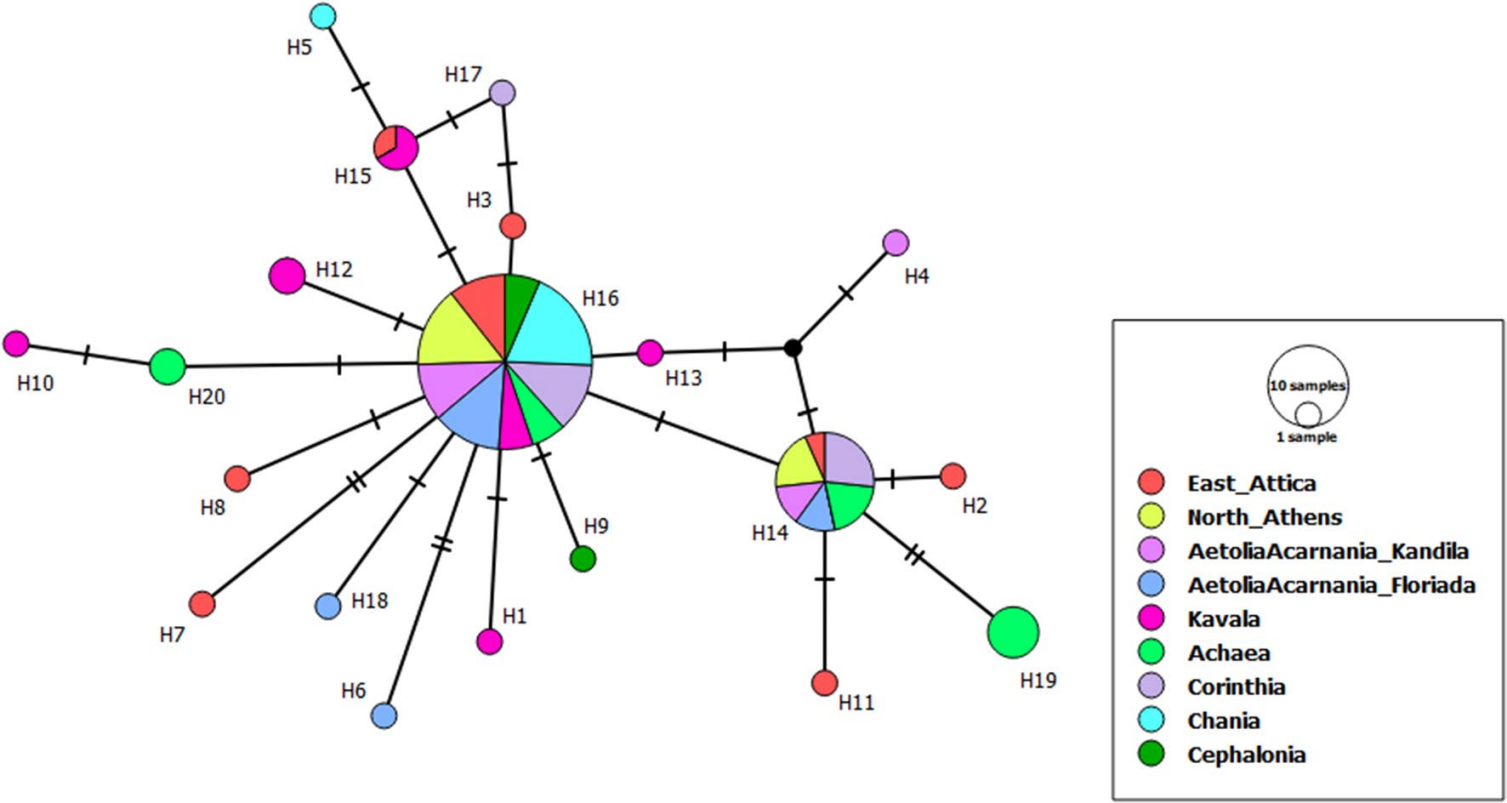
Haplotype *COI* network of *Philaenus spumarius* from Greece obtained from TCS analysis with PopART v.1.7 (http://popart.otago.ac.nz). Each colour indicates a different geographic area. Size of the circles represent the frequency of each haplotype among individuals. Hatch marks along the branches indicate the numbers of mutations and black dots represent unsampled and hypothetical haplotypes. Size of the lines are in proportion with the number of base substitutions.

**Figure 2:**
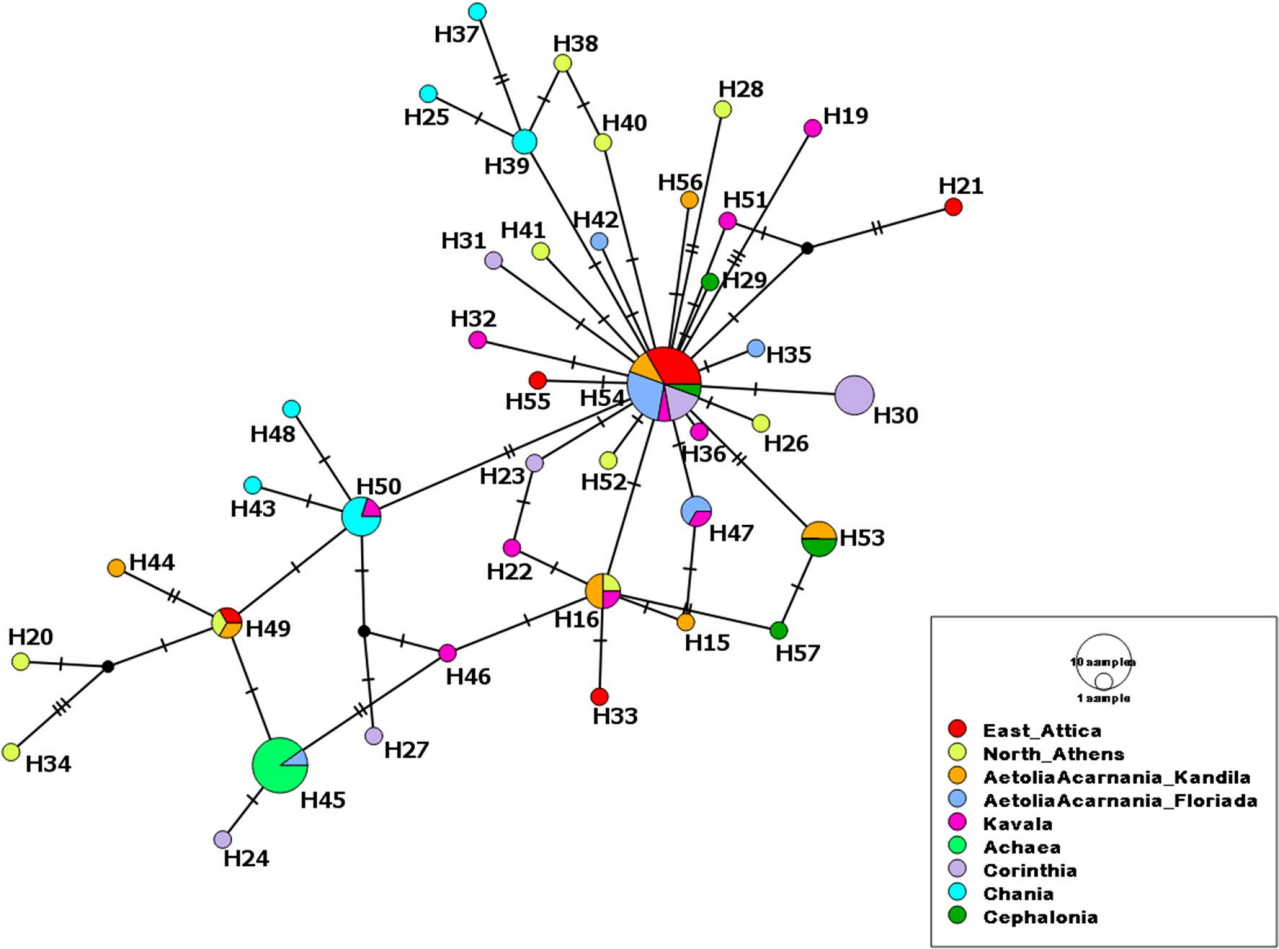
Haplotype *cytb* network of *Philaenus spumarius* from Greece obtained from TCS analysis with PopART v.1.7 (http://popart.otago.ac.nz). Each colour indicates a different geographic area. Size of the circles represent the frequency of each haplotype among individuals. Hatch marks along the branches indicate the numbers of mutations and black dots represent unsampled and hypothetical haplotypes. Size of the lines are in proportion with the number of base substitutions.

Sequences of *COI*, *cytb* and *ITS2* resulted in alignments of 462, 778, and 636 bp, respectively. The most variable populations were found to be the population from East Attica as well as the one from Kavala (North Greece) for the *COI* gene, with the latter being also variable for the *cytb* gene together with the population from North Athens. Most of the haplotypes occurred only once (14, 32 and 2 haplotypes for *COI*, *cytb* and *ITS2*, respectively) (Figs. 1 and 2, Supplementary Table S1, Supplementary Fig. S1). Haplotype PSp_CYTB_H54 of the *cytb* gene (Table 1 and Supplementary Table S1), was found in almost all examined Greek geographical sampling areas.

*COI* haplotypes presented 99.13–99.78% resemblance with AY63040 complete mitochondrial genome of *P. spumarius*^24^. All 20 haplotypes of *COI* gene presented here (PSp_COI_H1—PSp_COI_H20), have not been described before in previous studies. Among the 41 haplotypes of *cytb* found among Greek populations of the insect, PSp_CYTB_15 and PSp_CYTB_16 were shared with individuals from Spain and individuals from Turkey which belong to the SW (Supplementary Tables S1 and S3)^14,25^. The rest 39 haplotypes were detected for the first time and had not been previously described (Supplementary Table S1).

According to the *COI* haplotype network, based on the TCS algorithm, Greek populations of *P. spumarius* are divided in two main groups which however do not show any sign of segregation based on the geographical distribution of the populations. Several haplotypes derived from the most common haplotype of the first group PSp_COI_H16, and of the second, PSp_COI_H14, which differed by maximum two mutational steps, were shared between populations from all over Greece (Fig. 1 and Supplementary Table S1).

Names of the *cytb* haplotypes of *P. spumarius* were adjusted to the pattern and nomenclature described in Rodrigues et al.^14^. Haplotypes PSp_CYTB_H19 – PSp_CYTB_H57, obtained from Greek individuals, are described for the first time (Supplementary Table S1). Similar to the haplotype COI network, the haplotype cytb network (Fig. 2) consisting the Greek haplotypes obtained in the present study, separated the Greek populations in two clades, though without any indication of genetic structure based on their geographical origin. Most of the haplotypes were derived from the major haplotype PSp_CYTB_54 (Fig. 2).

**Figure 3:**
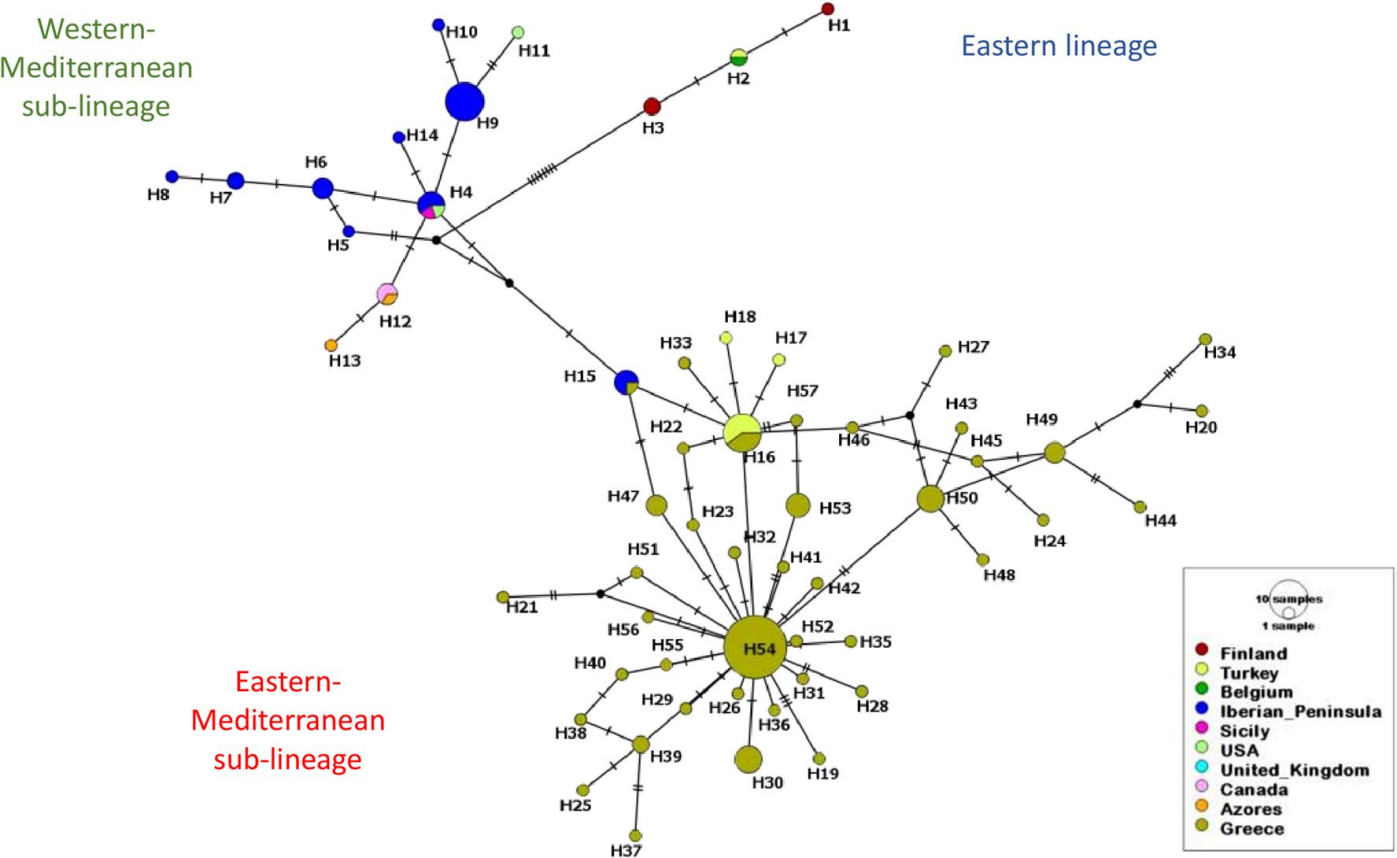
Haplotype *cytb* network of *Philaenus spumarius* from Greece and selected haplotypes found in rest of the species range. Each colour indicates a different geographic area. Size of the circles represent the frequency of each haplotype among individuals. Hatch marks along the branches indicate the numbers of mutations and black dots represent unsampled and hypothetical haplotypes. Size of the lines are in proportion with the number of base substitutions.

**Figure 4:**
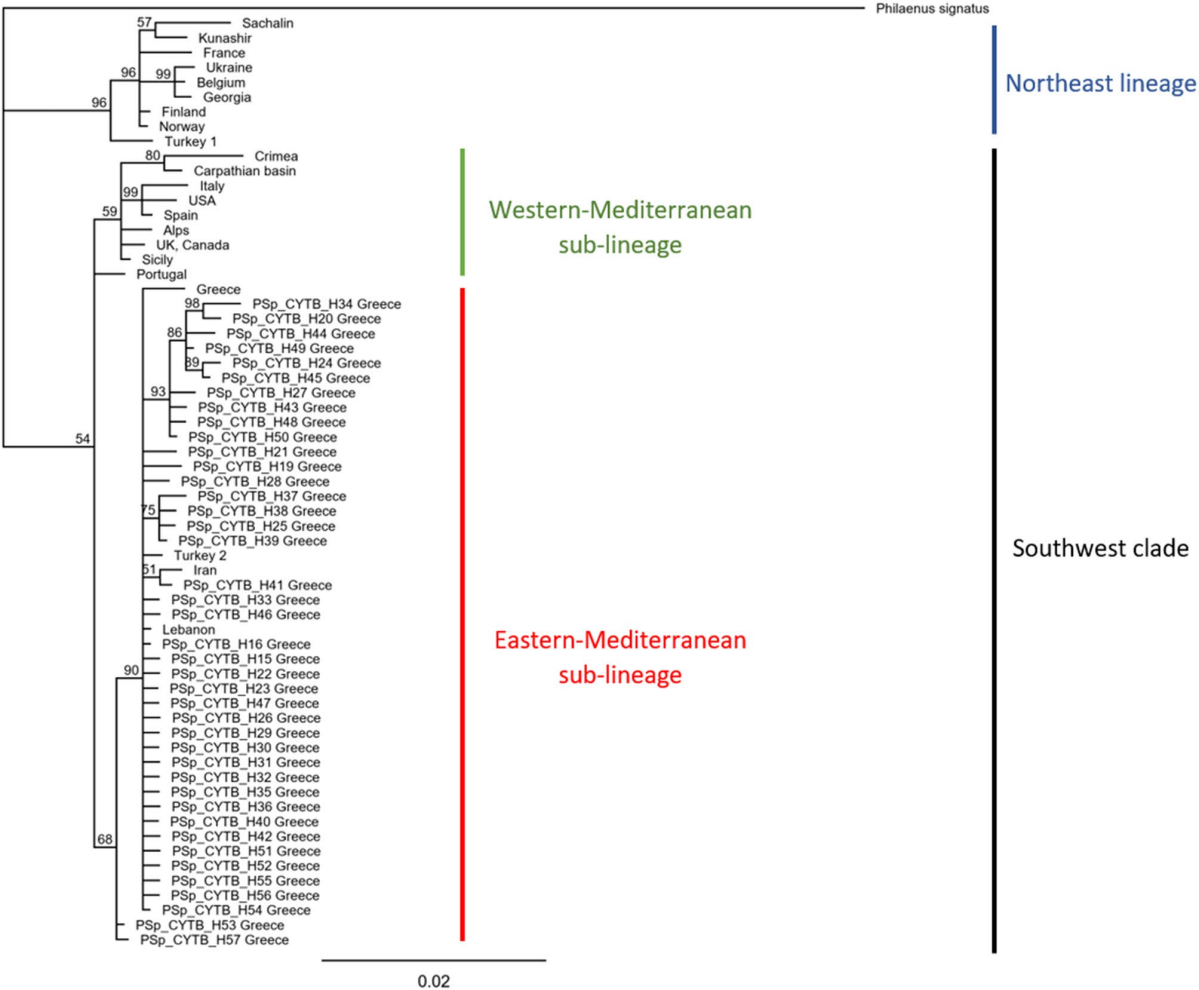
Phylogenetic reconstruction of *Philaenus spumarius* obtained from the analysis of *cytb* sequences. Available sequences of *cytb* from *P. spumarius* populations from distinct locations were included. Numbers above lines indicate posterior probabilities (%) of Bayesian Inference (only when above 50%). *P. signatus* was used as outgroup.

According to the results of both phylogenetic tree and haplotype network for the *cytb* gene constructed using individuals from different geographical regions of the world, *P. spumarius* was divided in two main clades (Southwest, SW and Northeast, NE), as has been proposed in previous studies (Figs. 3 and 4)^13,15,25^. All Greek *P. spumarius* individuals belonged to the Southwest clade, and particularly to the eastern-Mediterranean sub-lineage with the closest genotypes found in Iran, Turkey SW clade and Lebanon (Figs. 3 and 4).

**Figure 5:**
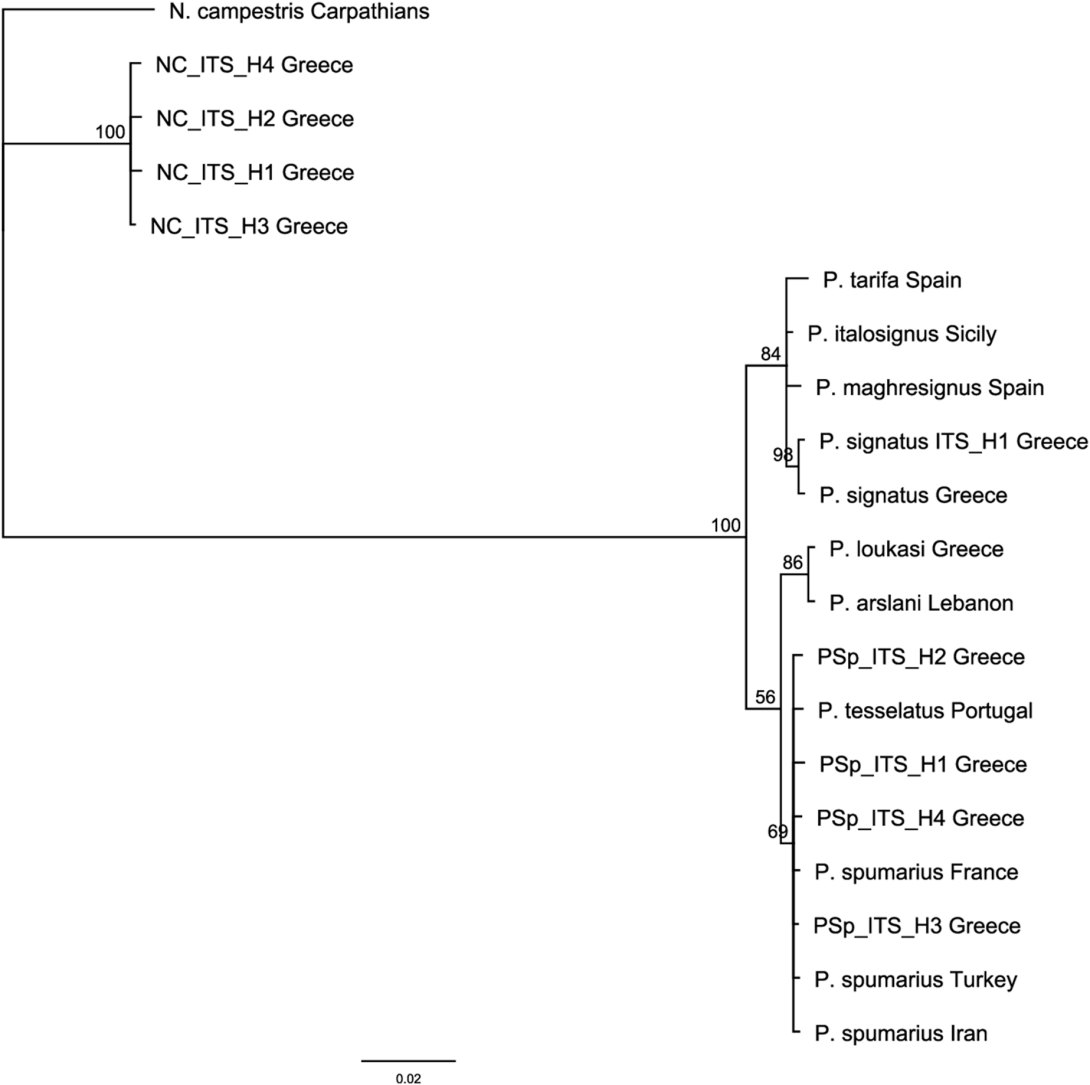
Phylogenetic reconstruction of different species of *Philaenus* and *Neophilaenus* obtained from the analysis of *ITS2* sequences. Bayesian Inference posterior probabilities (%) are shown next to the branches. Haplotypes from the three insect species detected in this study, are indicated in bold. *N. campestris* was used as outgroup.

Among the four haplotypes detected in this study for the ITS2 molecular marker (PSp_ITS_H1—PSp_ITS_H4), only the haplotype PSp_ITS_H3 was identical (100% identity in the overlapping fragment of 498 bp) with the already published sequences KP410337, KP410338 and HQ444290 from Turkey, Iran and Spain, respectively (Supplementary Table S3)^25^. The haplotype ITS2 network (Supplementary Fig. S1) constructed, displayed the single dominant haplotype PSp_ITS_H3 with the three remaining haplotypes being related to it. Phylogenetic analysis of *ITS2* sequences from different species of *Philaenus* (obtained Greek haplotypes of *P. spumarius* and *P. signatus* and already published ones from the same and from other species of the genus) and *N. campestris* indicated the well supported monophyly of the genus *Philaenus*, as has been already proposed by Maryańska—Nadachowska and co-workers^11^ (Fig. 5). However, the phylogenetic relationships between the *Philaenus* species were not determined (low posterior probabilities), probably due to the low polymorphism detected within the *ITS2* sequences.

### Genetic structure of Greek populations of *P. signatus*

Sequences of the *COI*, *cytb* and *ITS2* fragments resulted in 472, 780 and 645 bp, respectively. The most variable populations were found to be from Cephalonia_Livadi and Corinthia with three and five haplotypes for the *COI* and *cytb* gene, respectively (Table 1 and Supplementary Table S1). All individuals examined had the same *ITS2* haplotype (PS_ITS_H1), as the amplified fragment gave sequences that were identical in all studied individuals (Table 1 and Supplementary Table S1).

Among the published sequences of *P. signatus* in public databases, only two of them exist for COI (FJ516390) and ITS2 (FJ560710) molecular markers^11^ (Supplementary Table S3). Both of them belong to the same individual collected from Greece. Between the three haplotypes of *P. signatus* for the *COI* molecular marker (PS_COI_H1—PS_COI_H3), presented in Greek populations of the insect and the one sequence available in public (FJ516390) (Supplementary Table S3)^11^, there were only a few point mutations. Comparison between the Greek *ITS2* haplotypes and the published one (FJ560710) (Supplementary Table S3), showed a 100% similarity in the overlapping fragment of 500 bp (98% posterior probabilities in the phylogenetic tree) (Fig. 5). Due to the very low polymorphism and the lack of available sequences in the databases, no further analysis was conducted for the ITS2 molecular marker.

The haplotype networks constructed for *cytb* (Supplementary Fig. S2) and *COI* genes (Supplementary Fig. S3), showed that almost all presented haplotypes (apart from PS_CYTB_H1, PS_CYTB_H2 and PS_CYTB_H3 which were present in separate populations) were shared between individuals from the three Regional Units and were separated by one or two mutational steps.

### Genetic structure of Greek populations of *N. campestris*

A total fragment of 453, 778 and 638 bp, was obtained for the COI, cytb and ITS2 molecular markers, respectively. The most variable populations were found to be those from Corinthia (COI and ITS2 molecular markers) and East Attica_Vravrona (cytb and ITS2 molecular markers). The one from Corinthia displayed 8 different out of the 13 total detected haplotypes and 3 different haplotypes out of the 4 total detected haplotypes for the molecular markers COI and ITS2, respectively, and the one from East Attica_Vravrona displayed 11 different out of the 23 total detected haplotypes for cytb and 3 different out of the 4 total detected haplotypes for ITS2 (Table 1 and Supplementary Table S1).

The haplotype ITS2 network constructed by using the obtained Greek haplotypes, resulted in the major haplotype NC_ITS_H3 and the remaining three being separated from it by one mutational step (Supplementary Fig. S8).

Regarding the COI genomic region, the only two available published sequences of the species collected from Corsica, displayed 100% identity compared to haplotype NC_COI_H10 (Supplementary Fig. S4) (overlapping fragment of 435 bp). The remaining 12 haplotypes found in Greek populations, are described here for the first time. In the haplotype COI network (Supplementary Fig. S5), haplotype NC_COI_H13 was being presented as the predominant one while most of the others (apart from NC_COI_H6, NC_COI_H10 and NC_COI_H12) were unique to the respective Regional Units.

The BI phylogenetic tree (Supplementary Fig. S6) based on the obtained sequences of the *cytb* mitochondrial marker, resulted in a well-supported monophyly of *N. campestris* individuals from Greece. All newly obtained haplotypes clustered together in a well-defined clade (100% posterior probabilities) with several sub clusters within (posterior probabilities > 73%). The *Neophilaenus* sp. sequence of the *cytb* gene (KP410339) (Supplementary Table S3)^25^, from a Turkish individual exhibited 100 identity with haplotype NC_CYTB_H8 in the overlapping fragment of 640 bp and was situated into the Greek *N. campestris* clade (Supplementary Fig. S6). The haplotype cytb network (Supplementary Fig. S7) constructed using the obtained Greek haplotypes, separated them in two clades, none of which displayed geographical signals. The first presented one major haplotype (NC_CYTB_H20) containing individuals from all Regional Units, from which all the other haplotypes of the clade derived, and the other presented eight different haplotypes most of which were shared between the different populations.

### Detection of secondary endosymbionts and characterization of *Wolbachia* strains

The experiments conducted for the investigation of the bacterial community showed that all populations of all species were associated with secondary endosymbionts. Among the different insect species and populations within the insect-species studied, the infection status and frequency of the endosymbionts varied significantly. The highest endosymbiont diversity was found in *P. spumarius*. Populations of *P. spumarius* harbored *Rickettsia*, *Arsenophonus*, *Hamiltonella* and *Wolbachia*. Populations of *P. signatus* harbored only *Rickettsia* and those of *N. campestris* harbored *Rickettsia*, *Hamiltonella* and *Wolbachia*. There was no evidence for the presence of *Cardinium* in any of the three examined insect species (Table 2).

The infection status of the two *Philaenus* species varied from 0 to 100%, and that of *N. campestris* from 0 to 95%. More specifically, *Rickettsia* showed the highest prevalence in all species, and a near fixation or complete fixation to some populations. The three insect species harbored *Rickettsia* in different frequencies (0–100%), apart from three populations of *P. spumarius* (Aetolia-Acarnania_Floriada, Aetolia-Acarnania_Kandila and North Athens). *Arsenophonus* was detected in low frequencies only in *P. spumarius*, in two populations (Aetolia-Akarnania_Floriada and Kavala) out of the seven tested (14.28 and 20%, respectively). Both *P. spumarius* and *N. campestris* harbored *Hamiltonella* in low to medium frequencies (4.76–36.36%), with the exception of three populations belonging to *P. spumarius* (East Attica, Cephalonia and Kavala). *Wolbachia* occurred in *P. spumarius* sporadically at low frequencies (4.35–15%), in four populations out of the nine, and in three out of the four populations of *N. campestris*, but in higher frequencies (19.04–85%) (Table 2).

Νo polymorphism was detected between the obtained bacterial sequences within the population for each one of the three insect species. Moreover, the same strain of *Rickettsia* was present in both *Philaenus* species, and the same strain of *Hamiltonella* was present in both *P. spumarius* and *N. campestris*. Sequences of the detected bacteria have been deposited in GenBank database (NCBI) under the Accession Numbers: MT434978 (*Arsenophonus* of *P. spumarius*), MT434976 (*Hamiltonella* of *P. spumarius*), MT434980 (*Hamiltonella* of *N. campestris*), MT434981 (*Rickettsia* of *N. campestris*), MT434979 (*Rickettsia* of *P. spumarius*) and MT434977 (*Rickettsia* of *P. signatus*).

The MLST approach applied for all individuals of *N. campestris* harboring *Wolbachia*, assigned each *Wolbachia* isolate to ST217. In the five bacterial genes used (*gatB, coxA, hcpA, ftsZ* and *fbaA*), no polymorphism was detected. The Accession Numbers in GenBank database (NCBI) for the five bacterial genes of *Wolbachia* are MT433023 for *gatB*, MT433022 for *coxA*, MT433024 for *hcpA*, MT433021 for *ftsZ* and MT433020 for *fbaA*.

**Figure 6:**
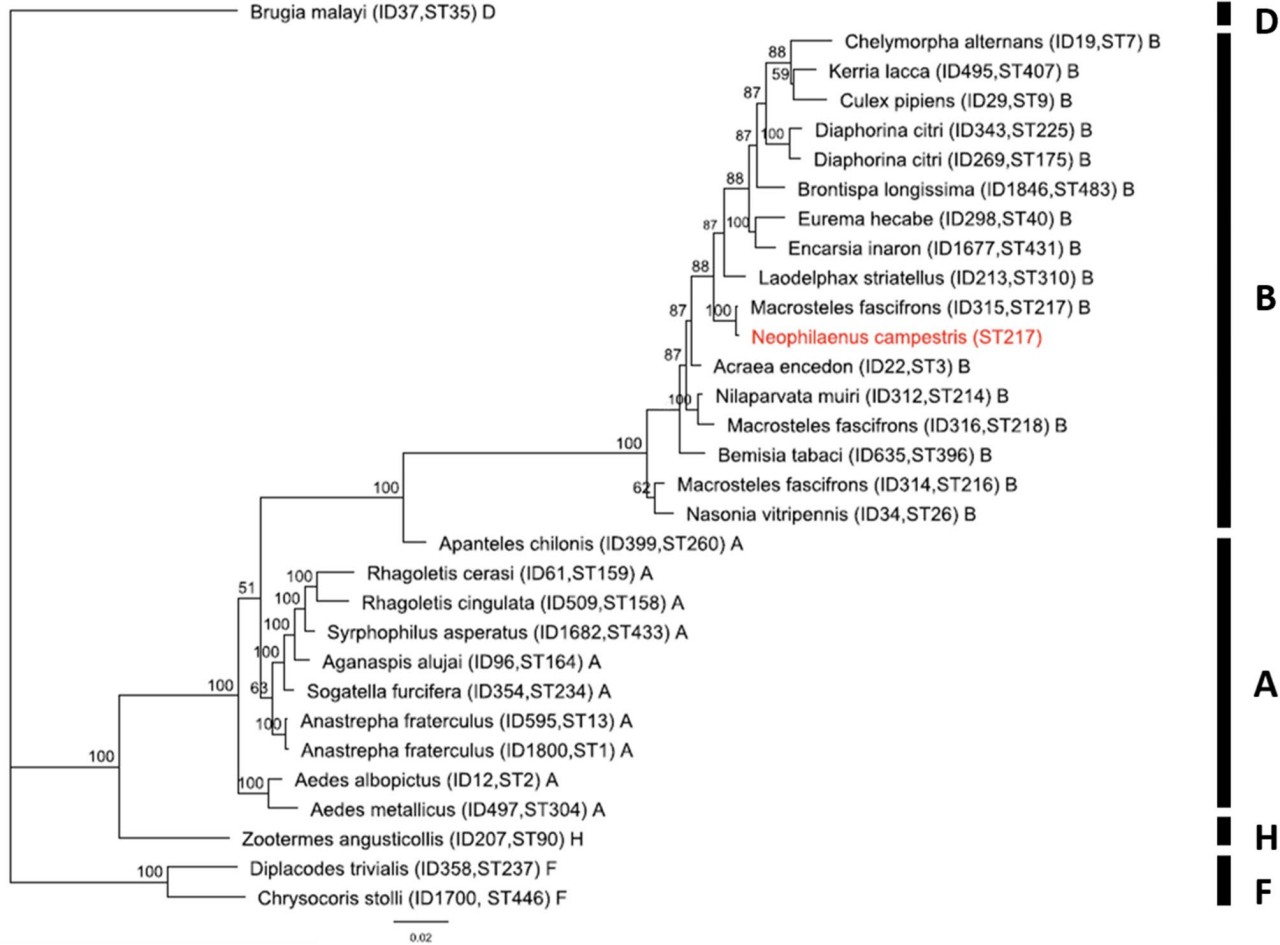
Bayesian Inference phylogeny based on the concatenated MLST data (2079 bp) of *Wolbachia* strains representing supergroups A. B. F and H. The strain of *Wolbachia* identified in this study is indicated in red color. Bayesian Inference posterior probabilities (%) are shown next to the branches. Capital letters indicate *Wolbachia* supergroups. The labels on the leaves correspond to *Wolbachia* strain names. Strains are characterized by the names of their host species, their IDs, the ST number from the MLST database and the supergroup they belong. *Wolbachia* supergroups are given to the right of the host species names. Supergroup D has been used as outgroup.

The MLST alignment analysis and the subsequent MLST-based BI tree with other known *Wolbachia*-mediated organisms separated the isolates used, into two major clusters (supergroup A and supergroup B) and supergroups H and F (supergroup D which infects only filarial nematodes^26^ was used as outgroup) (Fig. 6). In the phylogenetic tree, the *Wolbachia* strain from *N. campestris* individuals was clustered and classified as strain of the arthropod supergroup B, as it was clustered together with strains from other isolates of the same supergroup. The phylogeny revealed a high monophyletic relatedness of *N. campestris Wolbachia* strain to *Macrosteles fascifrons* (Hemiptera: Cicadellidae) and a relatedness with *Wolbachia*-mediated arthropods of the genera *Chelymorpha, Kerria, Culex, Diaphorina, Brontispa, Eurema, Encarsia, Laodelphax* and *Acraea*, belonging to supergroup B.

## Discussion

According to the results of the present study, the genetic diversity of the three species based on the haplotypes detected in relation to the number of individuals examined in the mitochondrial molecular markers (COI, cytb) was high. Conversely, in the ITS2 molecular marker, the variation observed, was rather low. It seemed to be highly conserved in all populations analyzed, and in *P. signatus* populations even monomorphic, since all studied individuals had the same ITS2 haplotype. Indeed, the general pattern of the nuclear sequence markers is often less variable than of mitochondrial markers, because of the combination of slower rates of evolution and the longer time that is necessary for lineage sorting^27,28^. ITS nuclear markers are mainly used in phylogenetic studies to infer the between-species variation. The phylogenetic tree constructed here, using ITS2 sequences obtained from the three species and already published ones from the same or related species, clearly separated the species of the genus *Philaenus* and *N*. *campestris*. Similar to previous studies^11,13^, *P. spumarius* and *P. tesselatus* fell into the same group separated from the groups containing the species *P. loukasi, P. signatus, P. arslani, P. maghresignus, P. italosignus* and *P. tarifa.*

The collected number of *P. signatus* Greek individuals was small, since this species was much less abundant than the other two insect species. Different ecological requirements (e.g., host plants’ range) are likely to affect population sizes between species that can consequently influence their distribution area and their genetic pattern^29^. In the present study, the examined insect species form different groups depending on larval food plant preferences. *Philaenus signatus’* larvae feed only on the lily *Asphodelus aestivus* Brotero, while *P. spumarius* and *N. campsteris* on various species of dicotyledons^30^. The low genetic diversity found in *P. signatus*, is consistent with the neutral theory that predict a relationship between the levels of genetic diversity and the population size and subsequently the mutation rates of genes^31^. In small populations, a mitochondrial haplotype will most probably be lost or gained since only few migration events would be sufficient to fix one mtDNA lineage in all populations^32,33^. Similarly, the patterns of polymorphism in the species *P. spumarius* and *N. campestris* which had adequate population sizes are also consistent with the neutral models of sequence evolution, since they had significantly higher levels of genetic diversity than the less common, *P. signatus*. Vector variation in efficiency transmission for plant pathogens has been demonstrated^34^. However, for *X. fastidiosa* transmission, the within insect species diversity is unknown and it is expected to be related to vectors’ behavior and ecology^34^. This is due to the fact that the bacterium is colonizing the cuticular surface of the insect-foregut, which is characterized as a conserved substrate among phloem-feeding insects and does not interact with live insect host cells^35^.

The indices of diversity (haplotype and nucleotide) of the three insect species were quite similar for COI and cytb molecular markers, comparing to the ITS2 molecular marker which presented lower genetic indices for all insect species. Populations of *P. spumarius* exhibited significant negative Tajima’s D and Fu’s Fs values for the molecular markers COI and cytb, which constitutes probably evidence for an excess of low frequency alleles, indicating a population expansion rather than positive selection^36,37^. Population expansion was confirmed also by the star-shaped haplotype networks and the high frequency of unique mutations^38–41^. Central haplotypes in networks have greater possibility of being ancestral, common and of broader geographic distribution while rare haplotypes are likely to be related to the common haplotypes and represent recent mutations^42^. In a rapidly growing population, the genetic variation will generally be accumulated and maintained and eventually will be beneficial for species’ success^43^. Due to the increased efficacy of selection in cleansing deleterious mutations and fixing advantageous ones, expanding populations often present faster evolution and increased population size^44,45^. When *P. spumarius* populations were grouped according to their geographical origin into North, Central and South Greece, the indices slightly changed since the groups of South and North Greece were less and more variable, respectively, than that of Central Greece. In general, the results of this study did not indicate a genetic structure among populations. When there are no geographic distances or barriers to limit the migration and gene flow between populations and to build up adaptive divergence between different habitats, population connectivity expedites and genetic differentiation between populations is decreased^46–48^.

Based on the mitochondrial divergence of the *cytb* gene, *P. spumarius* worldwide populations exhibited highly structured phylogeography^13,14,25,49^. The BI phylogenetic tree and the haplotype network presented a congruent topology. Populations were divided into the two previously described^13,15,49^, genetically highly distinct and diversified clades: a. the Southwest or western clade (SW) which was divided into the two subclades, the western Mediterranean and the eastern Mediterranean and b. the Northeast or eastern clade (NE). As it was expected, *P. spumarius* Greek individuals exhibited 41 haplotypes which were found to belong to the Southwest clade (SW) and more specifically to the eastern Mediterranean subclade, together with populations from the Balkans, and the Iberian and Anatolian peninsulas.

Bacterial endosymbionts in insects can influence various ecological traits of their hosts. The symbiotic associations represent evolutionary innovations and usually facilitate ecological adaptations and lineage diversification since closely related insect groups might have similar endosymbionts’ infections^18,50,51^. Moreover, endosymbionts can reduce the global genetic diversity or gene flow between populations within some insect species^52^. Effects of endosymbionts on insect hosts can vary from mutualism to pathogenicity. Endosymbionts supply the hosts with components essential for their survival, reproduction and development thus improving their physiology (resistance to abiotic stresses, adaptation to host plants and environments, pathogen transmission, insecticide resistance etc.) and invasiveness^18,52^.

Available molecular data regarding the obligatory endosymbionts of spittlebugs, exist only for the species *Clastoptera arizonana* (Clastopteridae) and *P. spumarius*. In both species the endosymbiont *Sulcia muelleri* is present together with the co-obligate endosymbiont *Candidatus* Zinderia insecticola^21^ and a *Sodalis* – like symbiont (most probably *S. glossinidius*)^23,53^. Among the different facultative endosymbionts, screening studies on the 16S *rRNA* gene on species of lineages of Cercopoidea and species from Cicadellidae and Membracidae, have shown the presence of *Wolbachia* (*Aphrophora quadrinotata*, *P. maghresignus*, *Cosmoscarta heros*), *Arsenophonus* (*Philaenarcys bilineata*) and *Rickettsia* (*N. lineatus*)^53^.

Our results showed that all populations of all species were associated with secondary endosymbionts. The low and intermediate frequencies, of all endosymbionts apart from *Rickettsia*, that most of the populations exhibited and the variation in the distribution, possibly means that they are not essential for their hosts, as has been suggested in symbiotic associations with different insect hosts^54^. On the other hand, this may be transient or may suggest equilibrium which changes depending on environmental and reproductive effects, metabolic costs, transmission efficiency etc.^55^. The bacterium *Wolbachia* is known to play a role as a mechanism on speciation processes of insect species, usually in populations belonging to distinct mitochondrial lineages, even though they belong to a common ancestral species^56^. Similar to the work of Lis et al.^15^, the signs of infection from *Wolbachia* in the Greek *P. spumarius* populations which belong to the SE clade (according to this study), were limited to a few populations of the species and with low percentages of infection.

The high abundance of the endosymbiont *Rickettsia* in almost all populations of *P. signatus* and *N. campestris* and in some of the populations of *P. spumarius* indicates mutualistic relationships between the endosymbiont and the insect species and may hint an important role of endosymbionts in their biology^57^. The high percentages of infection from endosymbionts combined with low mitochondrial genetic diversity, could possibly suggest a process of selective sweep. However, the examination of polymorphism of nuclear genes (in our case, the ITS2 nuclear region), which are not being affected by symbiont-driven selective sweeps, did not support this hypothesis. Likewise, considering the known effects of the bacterium on manipulating host reproduction by inducing male-killing and parthenogenesis (ladybird beetles, jewel beetles, eulophid wasps)^58,59^, the high abundance of the endosymbiont *Rickettsia*, deserves further investigation.

*Philaenus spumarius* and *N. campestris* are polyphagous species while *P. signatus* is monophagous^30^. The differences in the number of endosymbionts detected in the three species (*P. signatus* was found to be infected only by *Rickettsia*) may be correlated with the already proved relationships between symbiont occurrence and host plant specialization in other insect species^60–62^. Moreover, *P. signatus* plant-host dependent feeding behavior could affect its transmission competence as has been proved in the phloem-feeding insect species and phytoplasma vectors, *Euscelidius variegatus* (Kirschbaum) and *Empoasca decipiens* (Paoli)^63^. On the other hand, the high polyphagy of *P. spumarius* and *N. campestris* which may be related to the endosymbionts they carry, facilitates the spread of pathogens (in our case *X. fastidiosa*)^64,65^.

Τhe endosymbiont community may naturally determine the vectorial capacity and transmission of pathogenic agents and subsequently the spread of diseases either by impacting the interactions between the vector and its endosymbionts or by effecting the interactions between the endosymbionts and the pathogens within the vectors^64^. Examples of interactions between insect vectors, endosymbionts and phloem-limited phytopathogenic bacteria have already been described and include positive or negative correlations in acquisition or transmission of the phytopathogenic bacteria, reduction of symptoms, mutual exclusion, antagonistic activity against the pathogen and requirement of endosymbionts for transmission^66–71^.

Taking advantage of the insect-endosymbionts-pathogenic bacteria associations, strategies have been developed aiming to suppress the vector’s ability for transmission. Genetically modified endosymbionts or commensal bacteria could be used as media of delivering anti-pathogen molecules into vectors that block bacteria transmission^72^. This strategy, which is called paratransgenesis, has been tested for controlling Pierce’s disease in grapevines, caused by *X. fastidiosa*. Genetically modified forms of the bacterial endosymbionts *Alcaligenes xylosoxidans denitrificans* and *Pantoea agglomerans* of the sharpshooter *Homalodisca vitripennis* managed to disrupt pathogen transmission below detectable levels^73^.

Since populations of *N. campestris* presented the highest prevalence of the bacterium *Wolbachia*, a Multilocus Sequence Typing (MLST) analysis applied to characterize the bacterial strain of the species. The most recent classification of *Wolbachia*, discriminates 16 supergroups, which differ according to their hist biology and distribution^74^, from A to Q (except G)^75^. *Wolbachia* strains, which infect terrestrial arthropods, belong to the supergroups A and B^76^, while C and D supergroups are found only in filarial nematodes and E and H infect only springtails and termites^77,78^. According to our results *Wolbachia* of *N. campestris*, clustered together with that of *Macrosteles fascifrons* (Hemiptera: Cicadellidae) of supergroup B.

Taken together, this study contributes to our knowledge about population structure, genetic diversity and endosymbiotic composition of Greek populations of *P. spumarius, P. signatus* and *N. campestris*, three insect species known to be vectors of *X. fastidiosa*. Estimates of genetic diversity represent a valuable resource for biodiversity assessments and are increasingly used to guide conservation and management programs. High levels of genetic diversity enhance the potential for species for adaptive evolution and for maintenance and establishment to new environmental conditions. The combination of genetic data with knowledge on microbiota which could be the base for studying aspects concerning their role in the polyphagous nature of the insect pests, could be an important step in designing specific strategies on insect control and developing comprehensive integrated management strategies. The development of integrated strategies for the management of the xylem-sap feeding insects, vectors of the bacterium is consequentially a determinant for the control strategy of *X. fastidiosa.*

## Methods

### Sampling

**Figure 7:**
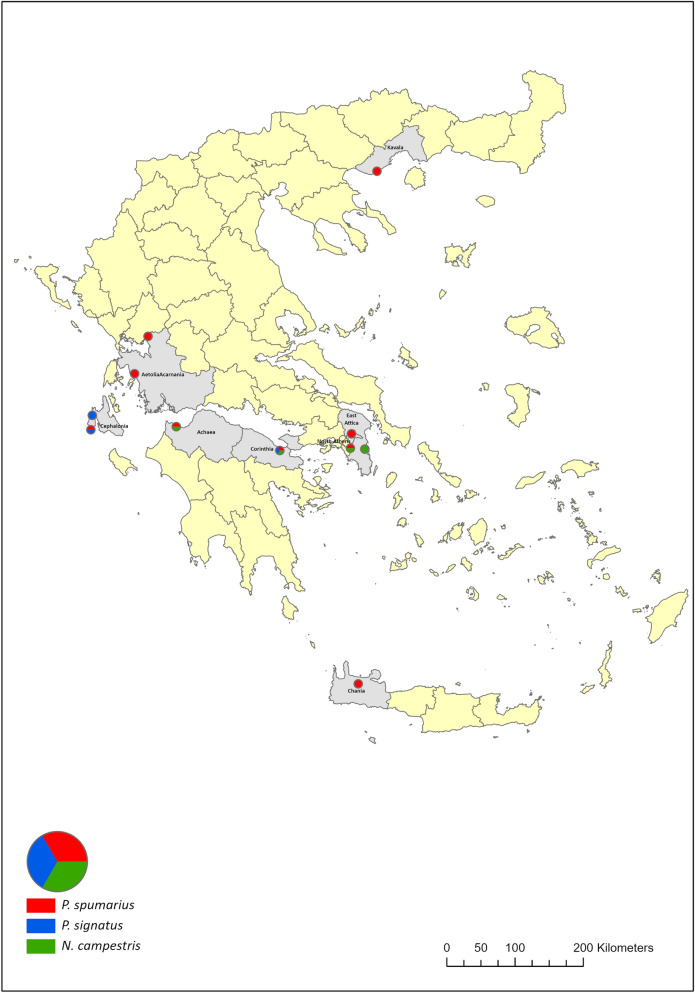
Collection sites of the analyzed populations of *Philaenus spumarius*, *P. signatus* and *Neophilaenus campestris*. The map has been generated with ArcGIS Pro v.2.5 software (https://pro.arcgis.com/en/pro-app/2.6/get-started/what-s-new-in-arcgis-pro-2-5.htm) (ESRI, RedLands, CA, USA).

During 2017 and 2018 a network of sites was selected in locations in central, south, north and west Greece to characterize xylem-sap feeder communities in relation to the threat by *X. fastidiosa* in non-affected regions. The sampling sites selected were olive orchards and were distributed in eight Regional Units (Table 3 and Fig. 7). Insect specimens were examined under microscope for species identification based on keys and illustrations^79–82^.

**Table 3:** Population codes of *Philaenus spumarius*, *P. signatus* and *Neophilaenus campestris* used for the analysis, including collection sites and total number of individuals from each population used for the genetic and endosymbiotic diversity.

Insect species	Population code	Regional Unit	Sampling site	Coordinates		No of individuals analyzed for the genetic diversity	No of individuals analyzed for the endosymbiotic diversity
				X	Y		
*P. spumarius*	570	East Attica	Spata	23,927425	37,954499	12	23
*P. spumarius*	545	North Athens	Kifissia	23,813044	38,082914	10	21
*P. spumarius*	540	Chania	Vatolakos	23,901411	35,446875	10	12
*P. spumarius*	539	Cephalonia	Damoulianata	20,381694	38,240269	5	5
*P. spumarius*	546	Aetolia-Acarnania	Kandila	20,95775	38,702508	10	20
*P. spumarius*	548	Aetolia-Acarnania	Floriada	21,13448	39,08487	10	20
*P. spumarius*	547	Kavala	Mirtofito	24,148215	40,751643	10	21
*P. spumarius*	642	Achaea	Lakopetra	21,506246	38,156479	12	20
*P. spumarius*	643	Corinthia	Ancient Korinthos	22,869247	37,909375	12	20
*P. signatus*	543	Cephalonia	Livadi	20,400683	38,273753	7	10
*P. signatus*	580	Cephalonia	Damoulianata	20,381694	38,240269	7	16
*P. signatus*	645	Corinthia	Ancient Korinthos	22,869247	37,909375	10	10
*N. campestris*	542	East Attica	Vravrona	23,988467	37,92317	17	20
*N. campestris*	541	East Attica	Spata	23,927425	37,954499	11	20
*N. campestris*	556	Corinthia	Ancient Korinthos	22,869247	37,909375	20	21
*N. campestris*	644	Achaea	Lakopetra	21,506246	38,156479	14	20

Individuals of *P. spumarius*, *P. signatus* and *N. campestris* were maintained in 98% ethanol and stored at − 20 °C for further molecular analysis. In total, 162 individuals from nine populations of *P. spumarius*, 36 individuals from three populations of *P. signatus* and 81 individuals from four populations of *N. campestris* were collected (Table 3).

### Extraction of genomic DNA

A total of 279 individuals belonging to the three species of spittlebugs, were used for the molecular analysis. Total genomic DNA was extracted from single individuals according to the European and Mediterranean Plant Protection Organization (EPPO) protocol for ctab-based (cetyltrimethyl ammonium bromide) DNA extraction for vectors^2^. Samples were stored at − 20 °C until use. Each extraction series contained positive and negative controls.

### PCR amplification, sequencing, and analysis of genetic diversity

All insect specimens were singly tested for the presence of the bacterium *X. fastidiosa*, using the molecular method proposed by Harper et al.^83^, as described in the EPPO protocol PM7/24 (4)^2^.

From the 279 individuals collected, 177 individuals were used in polymerase chain reactions (PCRs) that target partial fragments of mitochondrial and nuclear DNA. Fragments of the cytochrome oxidase subunit I (*COI*) gene, of the cytochrome b (*cytb*) gene and of the Internal Transcribed Spacer 2 (*ITS2*) region, were amplified with primers used in previous studies^11^ for the examination of the genetic variability of spittlebugs (Supplementary Table S4).

The PCR mastermix was prepared with 2 μL of 100 ng of gDNA and a mixture of 1 × Kapa Taq Buffer (Kapa Biosystems, Cape Town, South Africa), 0.2 mM dNTPs, 1.0 μM of each forward and reverse primer and 1 μL of Kapa Taq DNA polymerase (Kapa Biosystems, Cape Town, South Africa) in a final reaction volume of 25 μL. The thermocycling program was the same for the three molecular markers and included an initial denaturation of 3 min at 95 °C, followed by 35 cycles of 95 °C for 30 s, 50 °C for 1 min and 72 °C for 1 min and a final step of extension at 72 °C for 1 min. All reactions were run on a Verity 96-well Thermal Cycler (Applied Biosystems, Foster City, CA, USA). Suitable positive (morphologically identified individuals of the three insect species) and negative controls [Nuclease-Free water (ThermoFisher, Invitrogen, Carlsbad, California)] were included in each PCR reaction to avoid false negative and false positive results.

Amplifications were confirmed by using 5 μL of the PCR products on 1.2% agarose gel electrophoresis, containing Midory Dye, Green Staining. The rest PCR product was purified with the NucleoFast 96 Vacuum Manifold Kit (Macherey–Nagel, Germany) according to the manufacturer’s protocol and instructions and it was forwarded to Macrogen Sequencing service (Amsterdam, The Netherlands) for automated sequencing in both directions using the primers used for the amplification of each fragment of the three molecular markers analyzed. The obtained sequences for the amplified fragments of the three molecular markers (COI, cytb and ITS2) were edited using the Geneious Prime 2020.0.4 (https://www.geneious.com/) and aligned using MUSCLE v.3.8.425^84,85^. All alignments were verified by eye. Single representative sequences from the amplified molecular markers for the three insect-species were aligned against other sequences from previous studies for the evaluation of the discrimination of the species through the BLAST algorithm of the National Center for Biotechnology Information (NCBI, http://www.ncbi.nlm.nih.gov).

### Analysis of cytochrome b

Two haplotype networks for studying the phylogeographic pattern and geographic distribution of *P. spumarius* were constructed. The first was based on the haplotypes obtained in this study and the ones obtained from Rodrigues et al.^14^ who used the same pair of primers for the amplification of the *cytb* gene fragment. The haplotypes from Rodrigues et al.^14^ were from several geographical areas of Europe, America, and Asia. The second haplotype network was created based only on the Greek haplotypes. All sequences were aligned using MUSCLE v.3.8.425^84,85^ and converted in the appropriate format using DnaSP v.6 software (http://www.ub.edu/dnasp/)^86^ for subsequent analysis. PopART v.1.7 (http://popart.otago.ac.nz)^87^ was used to visualize haplotype distribution among countries by implementing the TCS algorithm^88^ and by using as traits the number of individuals carrying specific haplotypes per geographical region.

Additional analysis of the *cytb* gene of *P. spumarius* was performed by constructing a phylogenetic tree containing: A. single representative sequences of the amplified fragment of *cytb*, from previous studies on *P. spumarius* (Supplementary Table S3)^13,14,25,49^ representing all available sampling geographical regions and obtained mitochondrial clades, and B. the obtained cytb sequences of *P. spumarius* from this study. Analysis was computed using the Bayesian Inference (BI) method performed in MRBAYES v.3.2.1^89^. The best fit model of sequence evolution (HKY, Hasegawa-Kishino-Yano) for the dataset of *P. spumarius* was estimated using MEGA v.10.0.5^90^ under the Akaike Information Criterion (AIC). Gamma distribution with four gamma categories was used for modeling the evolutionary rate differences among sites. Analysis was performed using Monte Carlo Markov Chain method, iterated for 1,000,000 generations, with the temperature set to 0.2, a sampling frequency of 200 generations and a burn-in of 10,000.

Data for *cytb* gene of *N. campestris* available in GenBank database, are limited. Therefore, the phylogenetic tree obtainedusing the BI methodology under the HKY (Hasegawa-Kishino-Yano) model described above, contained single representatives from each haplotype presented in this study and the two sequences of *Neophilaenu*s sp., available in GenBank database (Supplementary Table S3)^25^. In addition, a haplotype cytb network was constructed, by applying the TCS algorithm^88^ implemented in PopART v.1.7 (http://popart.otago.ac.nz)^87^ to the obtained cytb Greek haplotypes of the species *N. campestris* following the same methodology as described above.

For the *cytb* gene of *P. signatus* only one sequence is available in the public databases (Supplementary Table S3)^25^. To infer the phylogeny of the obtained cytb Greek haplotypes of the species, a haplotype network using the same methodology as described above, was created.

### Analysis of cytochrome oxidase I

In order to visualize the phylogeographic relationships between Greek populations of the species *P. spumarius*, *P. signatus* and *N. campestris*, three haplotype networks were inferred using the TCS algorithm^88^ implemented in PopART v.1.7 (http://popart.otago.ac.nz)^87^ following the procedure described in “Analysis of cytochrome b”. The geographic distribution of the detected haplotypes in the Greek populations collected from the different Regional Units of the country (Table 3), was used as trait and employed in building the haplotype network.

In addition, a phylogenetic tree containing the Greek COI haplotypes of *N. campestris* and available COI sequences of *Neophilaenus* sp. (Supplementary Table S3)^12,91^ was constructed using the BI method implemented in MRBAYES v.3.2.1^89^. Based on the Akaike Information Criterion (AIC) as implemented in MEGA v. 10.0.5^90^, the HKY (Hasegawa-Kishino-Yano) model was chosen as the most statistically appropriate model for the data. Under the selected model, the parameters were optimized, and BI analyses was performed as described in “Analysis of cytochrome b”.

### Analysis of internal transcribed spacer

Due to lack of *ITS2* deposited sequences in Genbank (NCBI), for the three species (four, one and one sequences for the *ITS2* region of *P. spumarius, P. signatus and N. campestris*, respectively)^11,25^ (Supplementary Table S3), one combined phylogenetic tree was constructed for all three species, including sequences from *N. campestris* and other *Philaenus* species from distinct collection areas (Supplementary Table S3). Jukes-Cantor model was determined as the appropriate nucleotide substitution under the Akaike Information Criterion (AIC), as implemented in MEGA v.10.0.5^90^. Genetic analysis using the BI approach, was computed using MRBAYES v.3.2.1^89^, following the procedure described in “Analysis of cytochrome b”.

To examine the relationships between the obtained Greek ITS2 haplotypes of the species *P. spumarius* and *N. campestris*, two separate haplotype ITS2 networks using the TCS analysis implemented in PopART v.1.7 (http://popart.otago.ac.nz)^87^, were constructed following the procedure described in “Analysis of cytochrome b”. *Philaenus signatus* Greek populations were excluded from further analysis because they were found to be essentially conserved in the ITS2 molecular marker.

### Diversity and genetic analysis

To measure the genetic divergence among and within the three insect species, standard genetic indices were calculated for the three molecular markers (except for the *ITS2* region of *P. signatus* which appeared to be monomorphic). In addition, the genetic divergence of *P. spumarius* populations was examined when arranging them, according to their sampling region, into the following groups: North Greece (Kavala), Central Greece (East Attica, North Athens, Cephalonia, Aetolia-Acarnania_Kandila, Aetolia-Acarnania_Floriada, Achaea, Corinthia) and South Greece (Chania). Number of haplotypes (H), haplotype diversity (Hd), nucleotide diversity (Pi), number of polymorphic sites (PS), average number of nucleotide differences (k) and singleton variable (SVS) and parsimony informative (PIS) sites, were assessed using several functions of the DnaSP v.6 software (http://www.ub.edu/dnasp/)^86^. Tajima’s D^36^ and Fu’s Fs^37^ neutrality tests were also computed.

### Insect populations used in the analysis of endosymbiotic diversity

For the characterization of the endosymbiotic bacterial community, up to 23 individuals from different populations of the three species were used, depending on the available number of the collected individuals (Table 3). In total, 161, 36 and 81 individuals of *P. spumarius*, *P. signatus* and *N. campestris*, respectively, were screened to extract information about the distribution and infection status of five secondary endosymbionts (*Wolbachia*, *Arsenophonus*, *Hamiltonella*, *Cardinium*, *Rickettsia*), known to be capable of influencing their hosts’ biology and reproduction.

### Amplification and sequencing of endosymbionts

Individuals were screened for endosymbiont infection using specific PCR primers targeting the 16S *rRNA* gene for *Hamiltonella*, *Cardinium* and *Rickettsia*, the 23S *rRNA* gene for *Arsenophonus* and the *ftsZ* gene for *Wolbachia*.

Four microliters of genomic DNA extract were used as template in 50 μl reactions contained 0.2 mM dNTPs, 1.0 μΜ of each primer, 1 μl Kapa Taq DNA polymerase and 1 × enzyme buffer (Kapa Biosystems, Cape Town, South Africa). PCRs were performed in a Verity 96-well Thermal Cycler (Applied Biosystems, Foster City, CA, USA) using the following conditions: initial denaturation at 95 °C for 3 min, followed by 35 cycles of 95 °C for 30 s, 52–58 °C (depending on primer pairs) (Supplementary Table S4) for 45 s, and 72 °C for 1 min; and a final step of extension at 72 °C for 10 min. The amplified products were loaded and visualized on an 1.2% agarose gel containing the Midori Green Nucleic Acid gel stain (NIPPON Genetics, Europe).

To verify the identity of the amplicons and to detect possible bacterial genetic diversity within species, a representative (five individuals from each insect species, found to be infected by the respective endosymbiont) number of PCR products were purified using the NucleoFast 96 PCR Vacuum Manifold Kit (Macherey–Nagel, Germany) according to the manufacturer ‘s instructions and sequenced in both directions with the primers used for the amplification (Macrogen, Amsterdam, The Netherlands).

The sequences obtained were analyzed using the software package Geneious Prime 2020.0.4 (https://www.geneious.com/) with default parameters, manually edited when necessary and aligned using MUSCLE v.3.8.425^84,85^. Obtained sequences were compared with those already published in the GenBank database with the BLAST tool of NCBI (NCBI, http://www.ncbi.nlm.nih.gov). Alignment of the obtained sequences for all detected endosymbionts with the same or most similar strains from other hosts harboring the endosymbionts, followed for a comparative analysis.

### MLST analysis of *Wolbachia*

Due to the high percentage of *Wolbachia* infection in *N. campestris* populations, the *Wolbachia*-positive individuals from each population of the species were further analyzed by Multi Locus Sequencing Typing (MLST), for characterizing the strains of the bacterium, using the sequences of conserved genes as molecular markers.

PCRs and sequencing of five different genes of *Wolbachia* (*gatB*, *coxA*, *hcpA*, *ftsZ* and *fbaA*) were carried out using the primers, protocols and procedures described at https://pubmlst.org/ Wolbachia/^92^ (Supplementary Table S4). A Verity 96-well Thermal Cycler (Applied Biosystems, Foster City, CA, USA) was used for PCRs. Success of amplification was verified by loading 5 μl of each reaction on an 1.2% agarose gel, stained with Midori Green Nucleic Acid gel stain (NIPPON Genetics, Europe). PCR products were purified using the NucleoFast 96 PCR Clean-up kit (Macherey–Nagel, Germany) and both strands were sequenced (Macrogen, Amsterdam, The Netherlands).

### Phylogenetic analysis of *Wolbachia*

Editing of the sequences was performed using the software package Geneious Prime 2020.0.4 (https://www.geneious.com/). The alignment was performed using MUSCLE v.3.8.425^84,85^, with default parameters and manually edited when necessary. The sequences of *gatB*, *coxA*, *hcpA*, *ftsZ* and *fbaA* genes were submitted to the PubMLST database for strain genotyping, generating a MLST allelic profile.

Furthermore, the obtained sequences of the MLST genes were compared with the use of BLAST tool of NCBI (NCBI, http://www.ncbi.nlm.nih.gov) to find similar/other hosts harboring the same or most similar *Wolbachia* strain. A dataset of 30 reference *Wolbachia* isolates with known STs (concatenated sequences) from the MLST database representing supergroups A, B, D, F and H, was retrieved and used in the comparative analysis.

The relationships between different *Wolbachia* strains were featured through the construction of phylogenetic tree. The phylogenetic analysis of the concatenated sequences of the MLST genes was performed with MRBAYES v.3.2.1^89^ based on Bayesian Inference (BI). The General Time Reversible (GTR) substitution model was used and the analysis was conducted as described in Section “Phylogenetic analysis of cytochrome b”.

## Supplementary Information

Supplementary Information 1.

Supplementary Information 2.

Supplementary Information 3.

Supplementary Information 4.

Supplementary Information 5.
