# Supplementary material for: Genetic and endosymbiotic diversity of Greek populations of Philaenus spumarius, Philaenus signatus and Neophilaenus campestris, vectors of Xylella fastidiosa
Source: Sci Rep. 2021 Feb 12;11:3752. doi: 10.1038/s41598-021-83109-z (PMC7881138; doi:10.1038/s41598-021-83109-z)
Supplement: Supplementary file 1 — Supplementary Information 1. [file 41598_2021_83109_MOESM1_ESM.pdf]

Supplementary Information

Article Title: Genetic and endosymbiotic diversity of Greek populations of *Philaenus spumarius*, *Philaenus signatus* and *Neophilaenus campestris*, vectors of *Xylella fastidiosa*

Authors' names: Despoina Ev. Kapantaidaki*, Spyridon Antonatos, Vasiliki Evangelou, Dimitrios P. Papachristos and Panagiotis Milonas

***Corresponding author:** Despoina Ev. Kapantaidaki, Department of Entomology and Agricultural Zoology, Benaki Phytopathological Institute, 8 St. Delta str., Kifissia, Attica, Greece

e-mail: d.kapantaidaki@bpi.gr

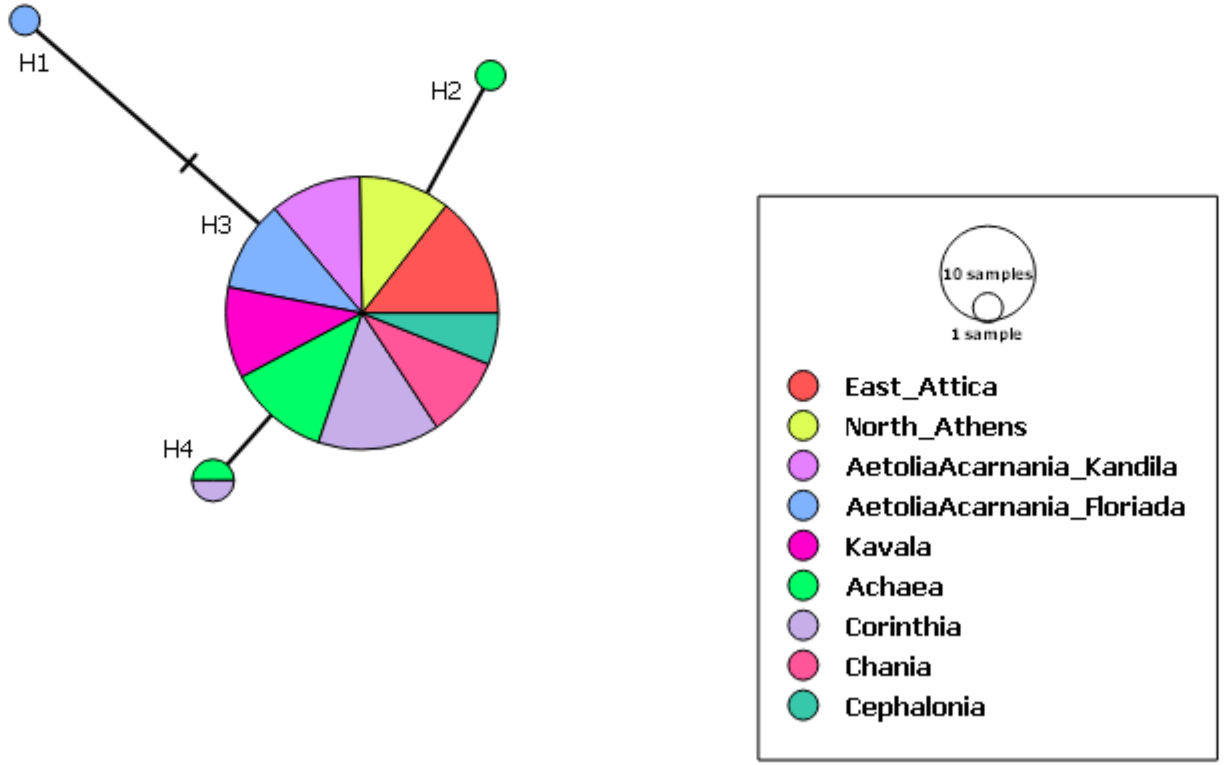

Supplementary Fig. S1. Haplotype ITS2 network of *Philaenus spumarius* from Greece obtained from TCS analysis with PopART v.1.7 (http://popart.otago.ac.nz). Each colour indicates a different geographic area. Size of the circles represent the frequency of each haplotype among individuals. Hatch marks along the branches indicate the numbers of mutations and black dots represent unsampled and hypothetical haplotypes. Size of the lines are in proportion with the number of base substitutions.

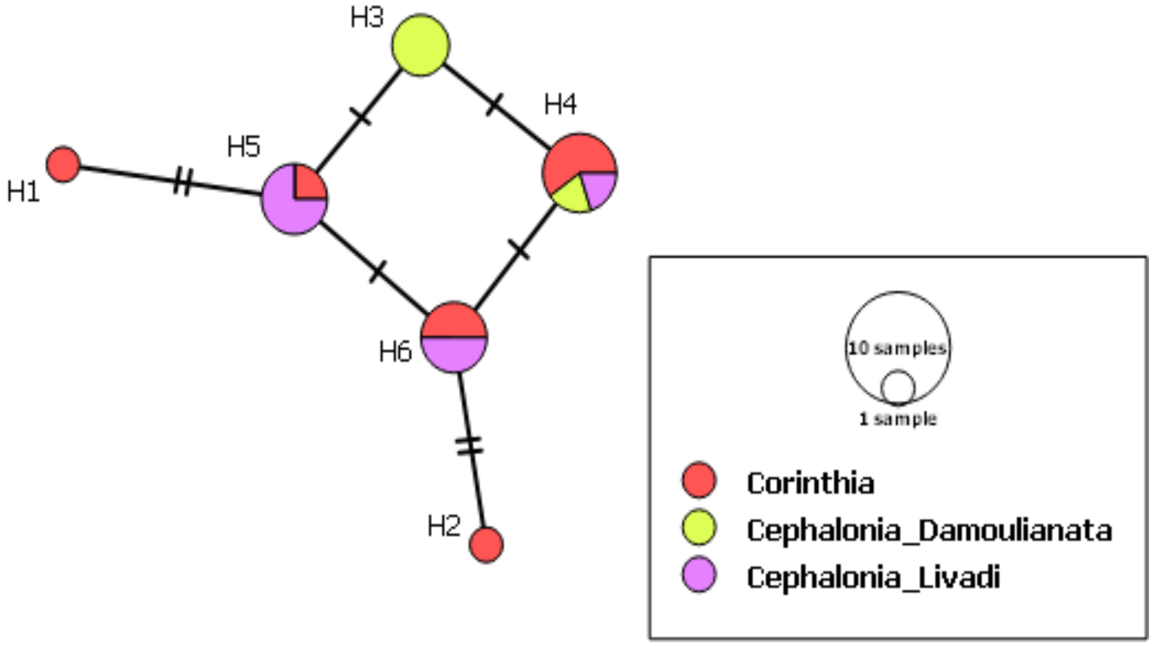

Supplementary Fig. S2. Haplotype cytb network of *Philaenus signatus* from Greece obtained from TCS analysis with PopART v.1.7 (http://popart.otago.ac.nz). Each colour indicates a different geographic area. Size of the circles represent the frequency of each haplotype among individuals. Hatch marks along the branches indicate the numbers of mutations and black dots represent unsampled and hypothetical haplotypes. Size of the lines are in proportion with the number of base substitutions.

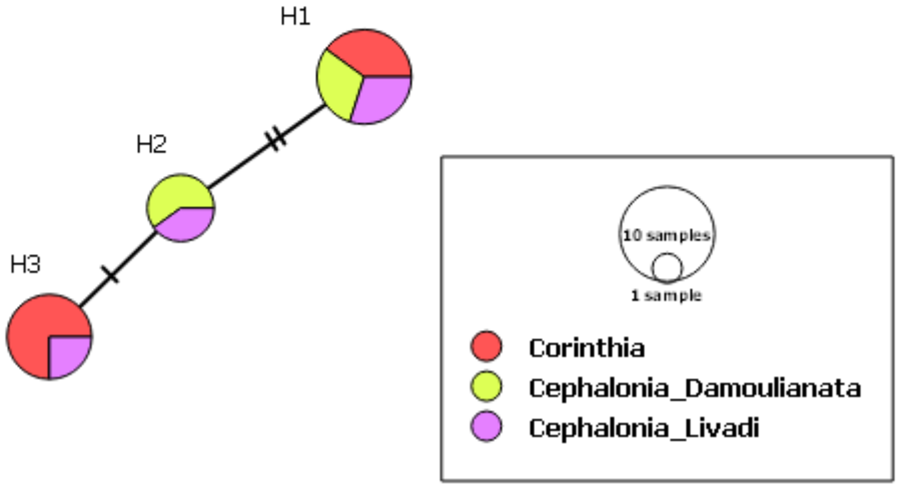

Supplementary Fig. S3. Haplotype COI network of *Philaenus signatus* from Greece obtained from TCS analysis with PopART v.1.7 (http://popart.otago.ac.nz). Each colour indicates a different geographic area. Size of the circles represent the frequency of each haplotype among individuals. Hatch marks along the branches indicate the numbers of mutations and black dots represent unsampled and hypothetical haplotypes. Size of the lines are in proportion with the number of base substitutions.

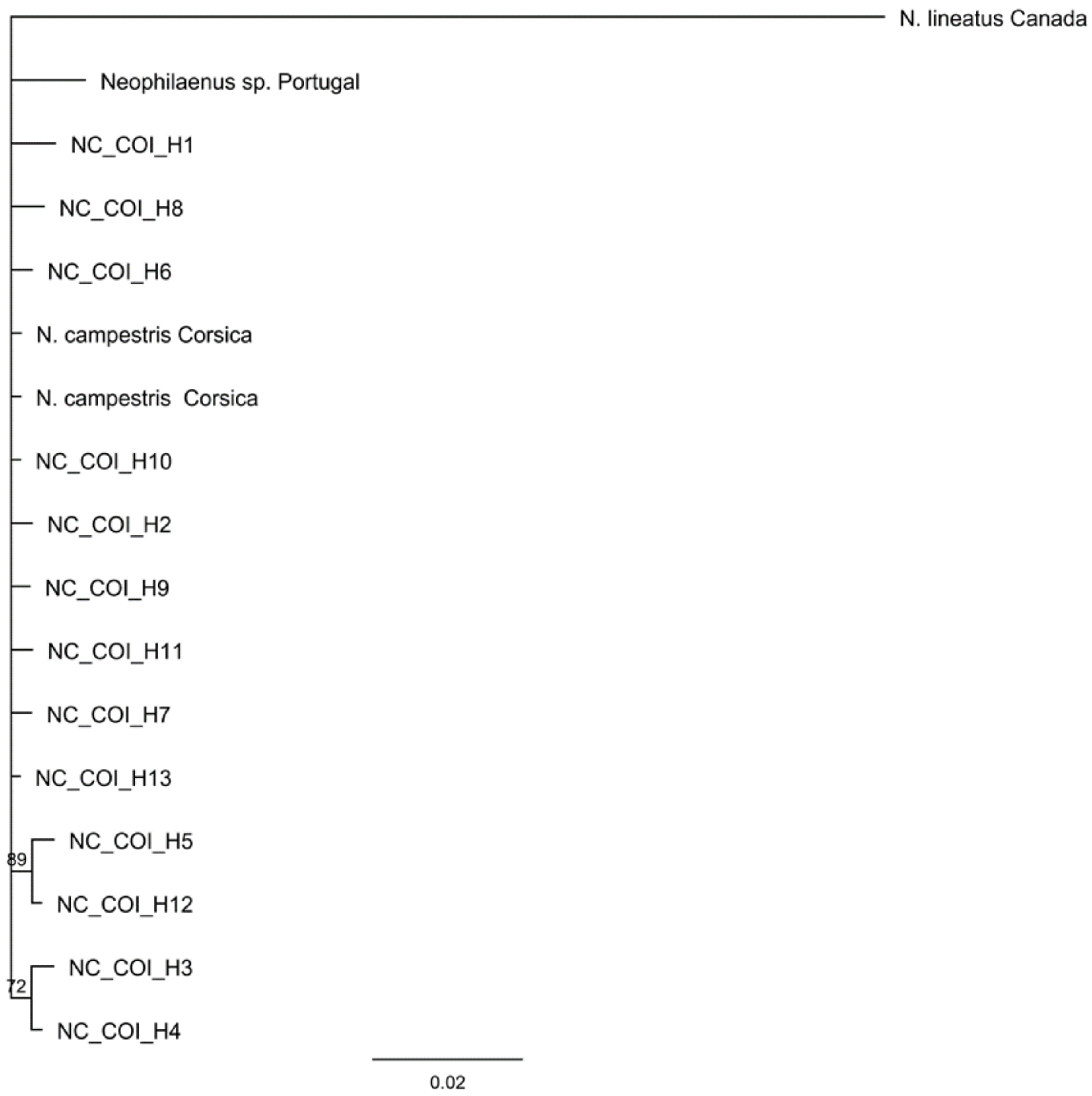

Supplementary Fig. S4. Phylogenetic reconstruction of *Neophilaenus campestris* obtained from the analysis of *COI* sequences. Bayesian inference posterior probabilities (%) are shown next to the branches. *N. lineatus* was used as outgroup.

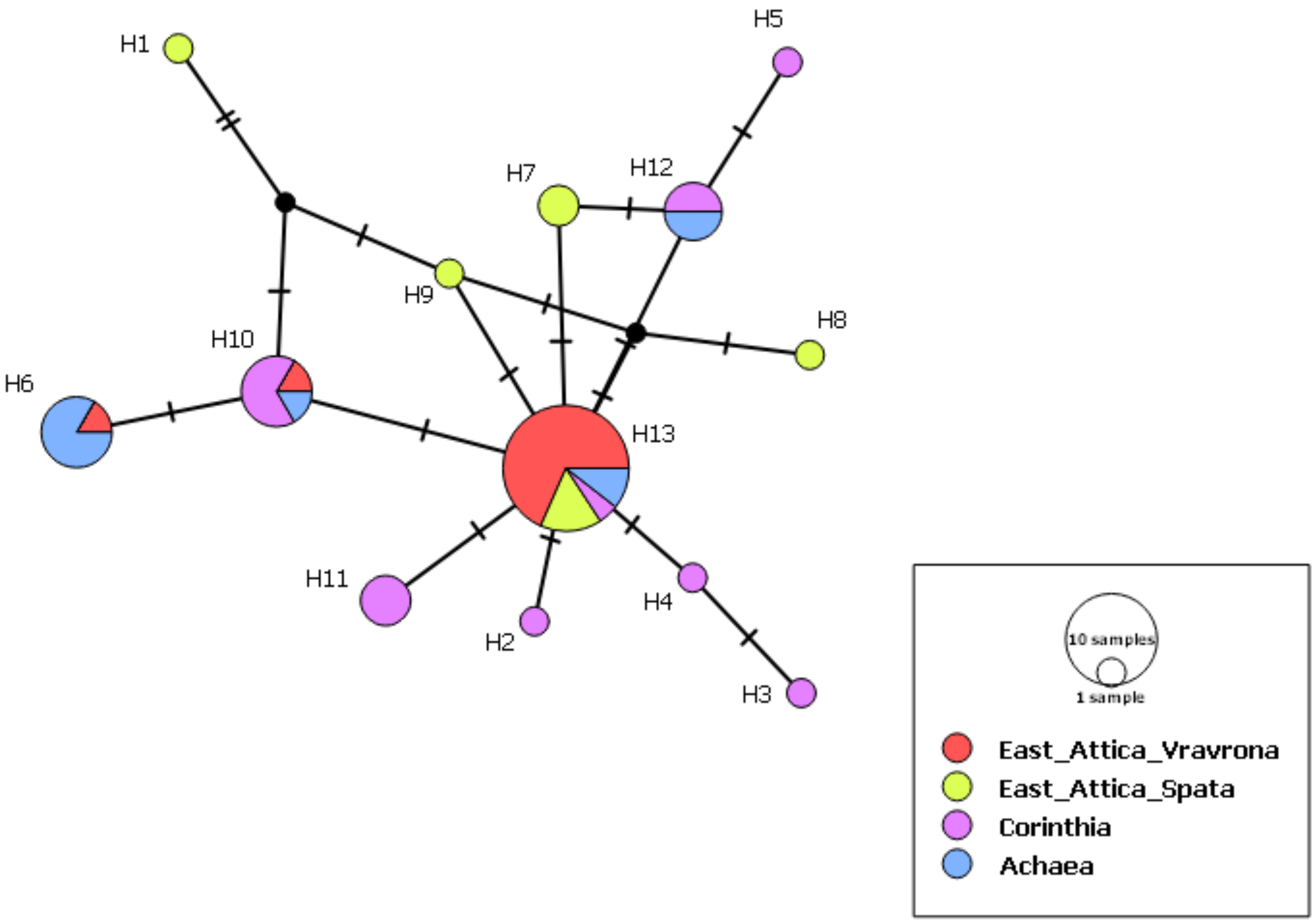

Supplementary Fig. S5. Haplotype COI network of *Neophilaenus campestris* from Greece obtained from TCS analysis with PopART v.1.7 (http://popart.otago.ac.nz). Each colour indicates a different geographic area. Size of the circles represent the frequency of each haplotype among individuals. Hatch marks along the branches indicate the numbers of mutations and black dots represent unsampled and hypothetical haplotypes. Size of the lines are in proportion with the number of base substitutions.

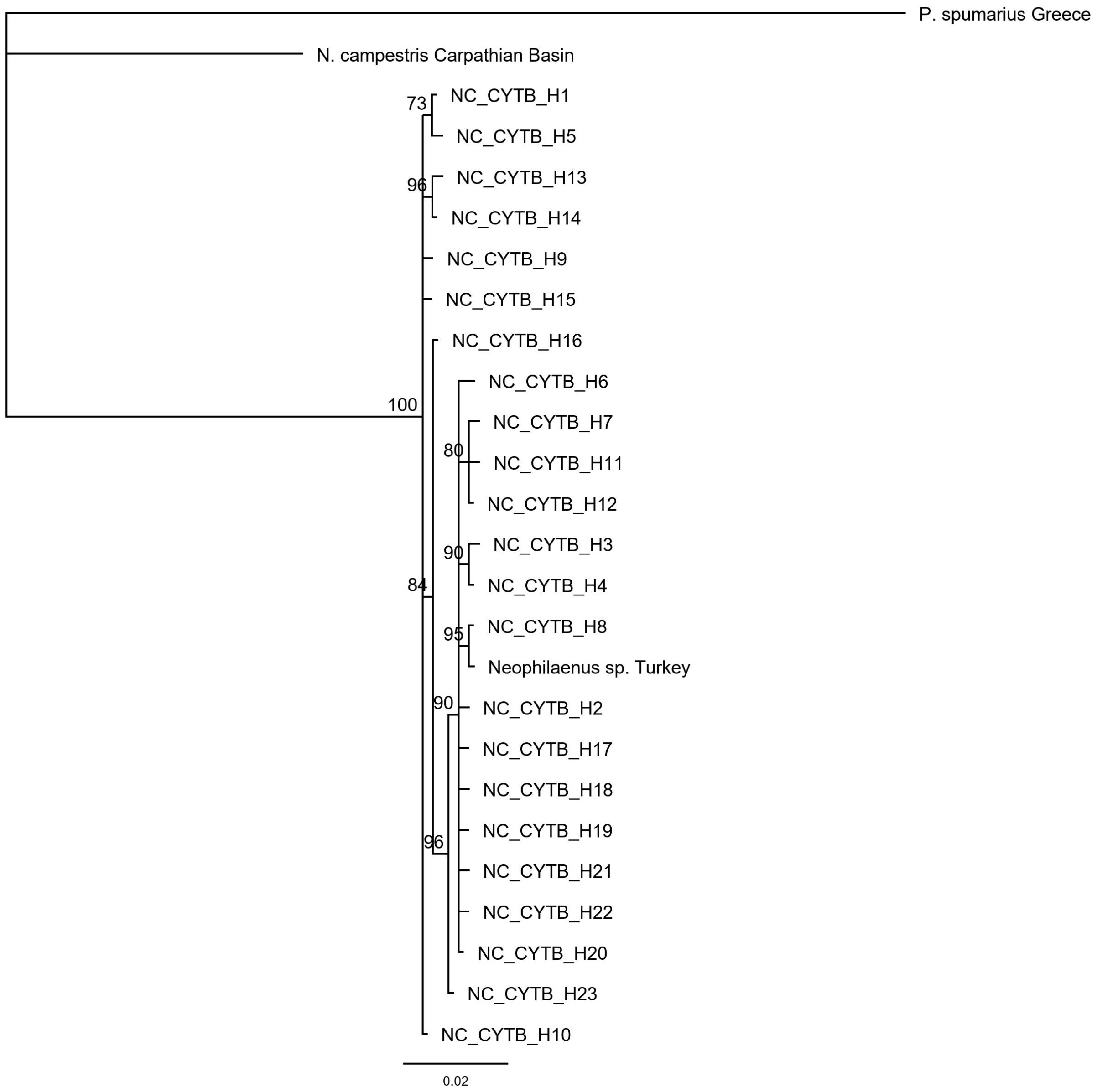

Supplementary Fig. S6. Phylogenetic reconstruction of *Neophilaenus campestris* obtained from the analysis of *cytb* sequences. Bayesian inference posterior probabilities (%) are shown next to the branches. *Philaenus spumarius* was used as outgroup.

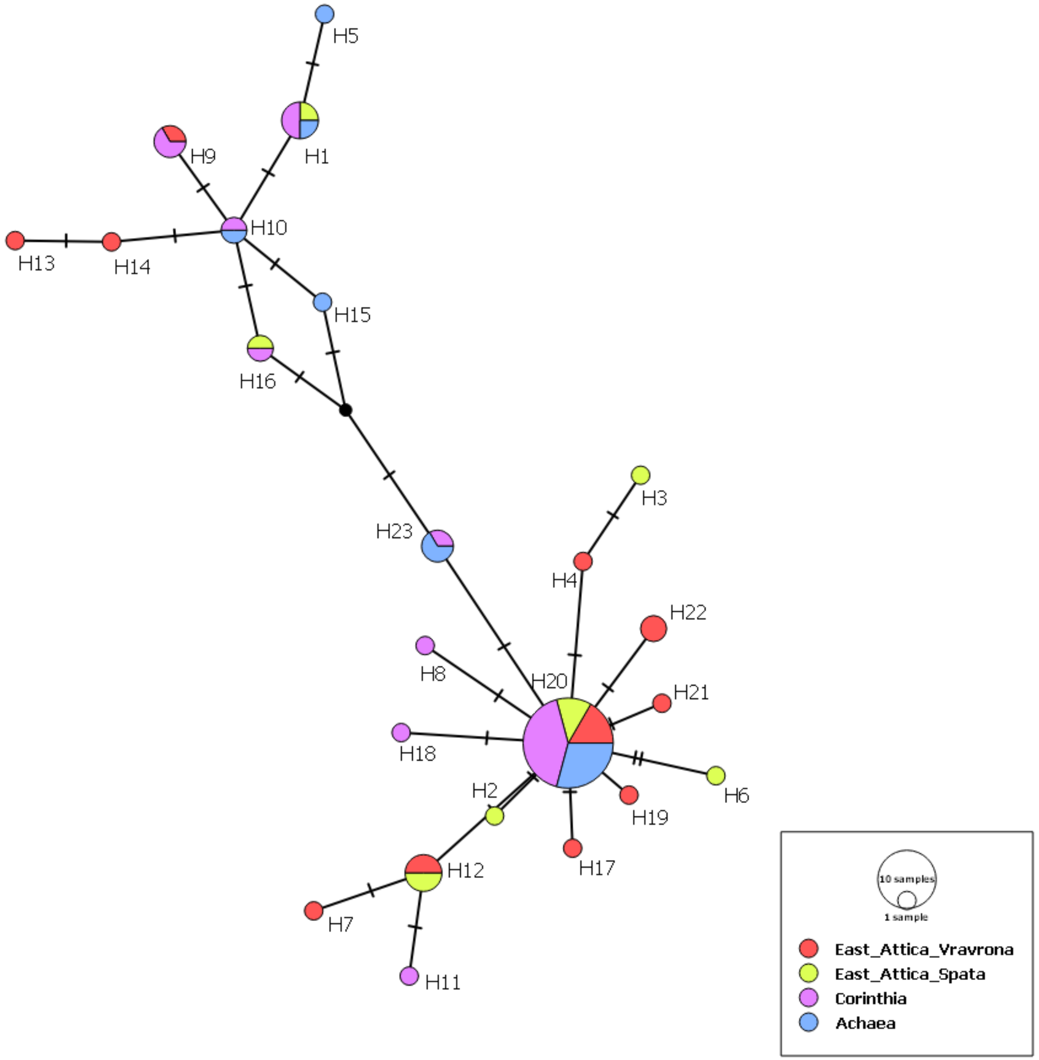

Supplementary Fig. S7. Haplotype cytb network of *Neophilaenus campestris* from Greece obtained from TCS analysis with PopART v.1.7 (http://popart.otago.ac.nz). Each colour indicates a different geographic area. Size of the circles represent the frequency of each haplotype among individuals. Hatch marks along the branches indicate the numbers of mutations and black dots represent unsampled and hypothetical haplotypes. Size of the lines are in proportion with the number of base substitutions.

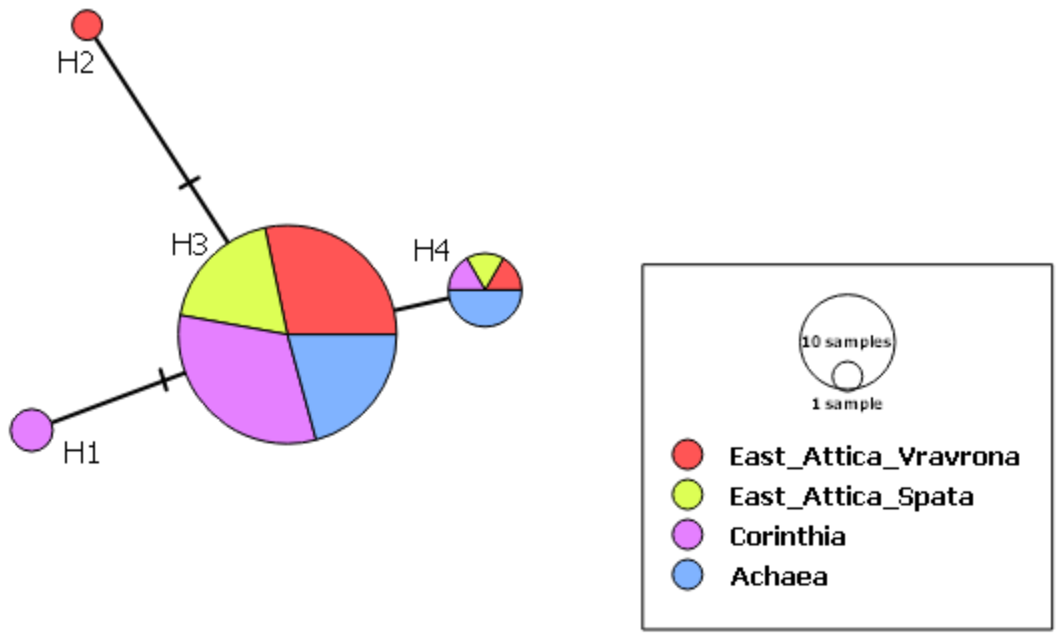

Supplementary Fig. S8. Haplotype ITS2 network of *Neophilaenus campestris* from Greece obtained from TCS analysis with PopART v.1.7 (http://popart.otago.ac.nz). Each colour indicates a different geographic area. Size of the circles represent the frequency of each haplotype among individuals. Hatch marks along the branches indicate the numbers of mutations and black dots represent unsampled and hypothetical haplotypes. Size of the lines are in proportion with the number of base substitutions.
